# Recent Progress in Active Mechanical Metamaterials and Construction Principles

**DOI:** 10.1002/advs.202102662

**Published:** 2021-10-29

**Authors:** Jixiang Qi, Zihao Chen, Peng Jiang, Wenxia Hu, Yonghuan Wang, Zeang Zhao, Xiaofei Cao, Shushan Zhang, Ran Tao, Ying Li, Daining Fang

**Affiliations:** ^1^ State Key Laboratory of Explosion Science and Technology Beijing Institute of Technology Beijing 100081 China; ^2^ Beijing Key Laboratory of Lightweight Multi‐functional Composite Materials and Structures Institute of Advanced Structure Technology Beijing Institute of Technology Beijing 100081 China

**Keywords:** active mechanical metamaterials, construction principles, engineering applications, multi functions, stimuli‐responsive materials

## Abstract

Active mechanical metamaterials (AMMs) (or smart mechanical metamaterials) that combine the configurations of mechanical metamaterials and the active control of stimuli‐responsive materials have been widely investigated in recent decades. The elaborate artificial microstructures of mechanical metamaterials and the stimulus response characteristics of smart materials both contribute to AMMs, making them achieve excellent properties beyond the conventional metamaterials. The micro and macro structures of the AMMs are designed based on structural construction principles such as, phase transition, strain mismatch, and mechanical instability. Considering the controllability and efficiency of the stimuli‐responsive materials, physical fields such as, the temperature, chemicals, light, electric current, magnetic field, and pressure have been adopted as the external stimuli in practice. In this paper, the frontier works and the latest progress in AMMs from the aspects of the mechanics and materials are reviewed. The functions and engineering applications of the AMMs are also discussed. Finally, existing issues and future perspectives in this field are briefly described. This review is expected to provide the basis and inspiration for the follow‐up research on AMMs.

## Graphical abstract

Active mechanical metamaterials (AMMs) are newly emerged metamaterials in recent decades. Due to the elaborately designed internal micro‐structures and stimuli‐responsive characteristics, AMMs have both the advantages of unusual mechanical properties of mechanical metamaterials and the smart actuation functions of active materials. This review reveals the construction principle of the AMMs and reports recent progress in this field.

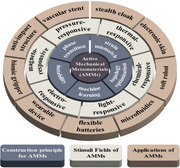

## 1 Introduction

By periodically arranging customized artificial microstructure units, mechanical metamaterials have extraordinary properties that do not exist in natural materials. Typical mechanical metamaterials include stiffness designable metamaterials,^[^ ^1^, ^2^ ^]^ pentamode metamaterials,^[^ ^3^, ^4^, ^5^, ^6^ ^]^ negative Poisson's ratio metamaterials,^[^ ^7^, ^8^, ^9^ ^]^ negative thermal expansion (NTE) metamaterials,^[^ ^10^, ^11^, ^12^ ^]^ and the origami or kirigami based metamaterials.^[^ ^13^, ^14^, ^15^, ^16^ ^]^ Benefitting from their superior properties, mechanical metamaterials have been used in various research and engineering fields. For example, the multi‐stable mechanical metamaterials were able to customize the stress–strain curves of multiple common materials like silicone foam composite, aluminum foam, and silk scaffold only through in situ switch.^[^ ^17^ ^]^

In recent years, stimuli‐responsive materials with unique properties and performances emerge rapidly, which provides new opportunities for the development of mechanical metamaterials. The most commonly used stimuli‐responsive materials include shape memory polymers (SMPs), liquid crystal elastomers (LCEs), hydrogels, and some other composites. If the mechanical metamaterials are reconstructed by replacing the conventional materials with stimuli‐responsive materials, they will be able to react to the stimuli of external physical fields, such as, heat,^[^ ^18^, ^19^, ^20^ ^]^ chemicals,^[^ ^21^, ^22^ ^]^ light field,^[^ ^23^, ^24^ ^]^ electric current,^[^ ^25^, ^26^ ^]^ magnetic field,^[^ ^27^, ^28^, ^29^ ^]^ and pressure action.^[^ ^30^, ^31^ ^]^ When stimulated, the metamaterials can automatically deform, make motions, and change their structural properties or functions according to external environments, which thus can be named active mechanical metamaterials (AMMs). To make full use of the unique advantages of different material systems, researchers will select the appropriate stimuli‐responsive materials according to different and application requirements. For example, LCEs with good thermal‐responsive characteristics often serve as actuators or artificial muscles, and the morphing of LCE productions could be remotely controlled by changing the ambient temperature.^[^ ^32^, ^33^ ^]^

Compared with the conventional mechanical metamaterials, AMMs added an additional dimension of time, and the structural performance becomes dynamically adjustable. Not only the artificial internal structures but also the stimuli‐responsive materials play a role during the functioning of AMMs. If implanting a set of elaborately designed energy transfer and storage mechanisms into the metamaterials, the metamaterials would behave “smart,” and realize various functions comparable to the activity of life‐like bodies. Based on this principle, a large number of new functions could be extended such as, active shape‐shifting,^[^ ^34^, ^35^, ^36^ ^]^ programmable mechanical properties,^[^ ^37^, ^38^, ^39^ ^]^ elastic wave propagation control,^[^ ^40^, ^41^ ^]^ and mobility.^[^ ^42^, ^43^, ^44^ ^]^ AMMs is an emerging subject that continues to develop with mechanical design and material science, and has huge application prospects in engineering and science. For one thing, the construction of the AMMs structures are based on the general mechanical principles. For another, the properties of the component stimuli‐responsive materials determine the functions and applicable fields of the AMMs. These two contents are of great importance for AMMs. But currently, reports considering both of these two aspects are limited. It is necessary to discuss the mechanical construction principles and the classifications based on the stimulus fields, along with the most advanced technology, research results, and engineering applications of AMMs.

**Figure 1:**
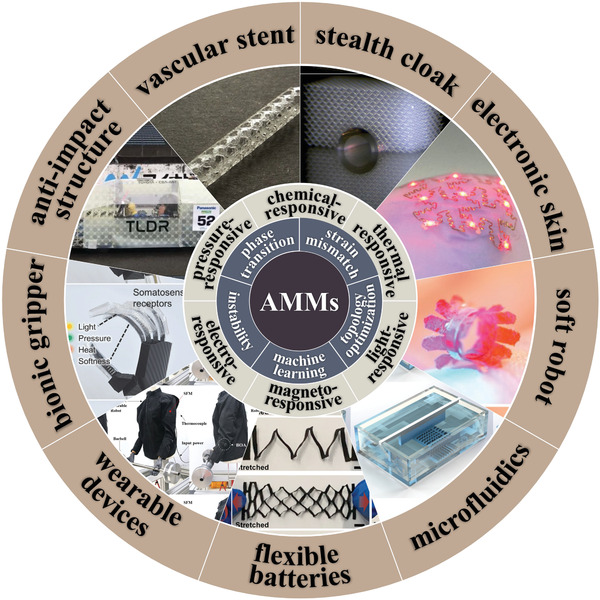
Summary of active mechanical metamaterials, their construction principles, classifications, and applications. Primary applications of AMMs are listed, including: a) Stealth cloak.^[^ ^48^ ^]^ Copyright 2014, Nature Publishing Group. b) Electronic skin.^[^ ^49^ ^]^ Copyright 2019, Wiley‐VCH. c) Soft robot.^[^ ^50^ ^]^ Copyright 2020, Wiley‐VCH. d) Microfluidics.^[^ ^28^ ^]^ Copyright 2020, Nature Publishing Group. e) Flexible batteries.^[^ ^51^ ^]^ Copyright, 2020, American Chemical Society. f) Wearable devices.^[^ ^52^ ^]^ Copyright 2019, Nature Publishing Group. g) Bionic gripper.^[^ ^53^ ^]^ Copyright 2020, Wiley‐VCH. h) Anti‐impact structure.^[^ ^46^ ^]^ Copyright 2020, American Association for the Advancement of Science. i) Vascular stent.^[^ ^54^ ^]^ Copyright 2018, Wiley‐VCH.

In this review, the discussion will be divided into three parts, as shown in **Figure** **1** (from inner to outside). We will start with the mechanical construction principles of active metamaterials, that is, 1) phase transition, 2) strain mismatch, 3) mechanical instability, 4) topology optimization, and 5) machine learning. The second part classifies AMMs according to stimulus fields and comprehensively reviews the research progress of each branch direction, that is, 1) thermal‐responsive AMMs, 2) chemical‐responsive active AMMs, 3) light‐responsive AMMs, 4) electro‐responsive AMMs, 5) magneto‐responsive AMMs, and 6) pressure‐responsive AMMs. Of course, these do not represent all types of stimuli‐responsive materials. With the development of material science and physics, new alternative stimuli‐responsive materials would emerge in the future. In the third part, we summarize several representative functions of mechanical metamaterials and their practical applications. They can be used in various fields such as miniaturized systems,^[^ ^45^ ^]^ huge machinery,^[^ ^46^ ^]^ and aerospace structures.^[^ ^47^ ^]^ Compared with previous mechanical metamaterials, AMMs possess more design flexibilities, pre‐stress design realizability, mechanical properties programmability, multi‐stimulus fields coupling driving characteristics, and so on. It can be expected that AMMs will play a more important role in manufacturing and human life.

## 2 Construction Principle for Active Mechanical Metamaterials

### 2.1 Mechanical Construction Principle

#### 2.1.1 Phase Transition

Phase transition refers to the changes of the material microstructures when the external field (such as, temperature, external force) continuously applies to the material until a specific condition, and the effect is always accompanied by the physical and mechanical changes. The most common phase transition phenomenon is the mutual transformation of materials between the solid phase, liquid phase, and gas phase. In the process of phase transition, the molecular level interactions between each part of the constituents make the whole material undergo a series of changes in properties or deformations. Therefore, the phase transition phenomenon is essentially the reconfiguration of the microstructures inside the material. And the changes in mechanical properties and shape memory effect caused by phase transition effect could be utilized as the construction method for a large class of AMMs. SMPs, LCEs, hydrogels, and magneto‐responsive materials based on the principle of phase transition are commonly used in AMMs. In this section, we would focus on the mechanical construction principle and take the most representative thermal‐responsive SMPs for example to review the progress.

**Figure 2:**
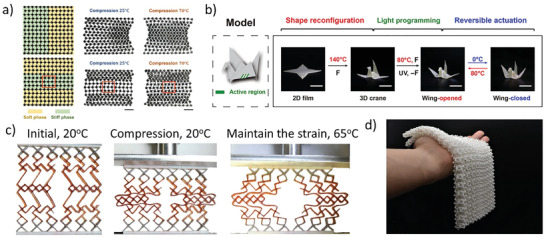
Metamaterials based on phase transition principle. a) Compression behaviors of the 2D metamaterials with the different material layout at 25 and 70 °C.^[^ ^61^ ^]^ Copyright 2019, American Physical Society. b) Reversible actuation of the bird‐like robot.^[^ ^62^ ^]^ Copyright 2018, American Association for the Advancement of Science. c) The programmable deformation of the smart window under temperature changing.^[^ ^18^ ^]^ Copyright 2018, Wiley‐VCH. d) Two layers of the chain‐mail fabrics in the soft state.^[^ ^67^ ^]^ Copyright 2021, Nature Publishing Group.

SMPs are active materials that can change their shapes from the original shape to a temporary shape when exposed to a thermal stimulus.^[^ ^55^ ^]^ In 2006, Liu et al.^[^ ^56^ ^]^ simplified the classic thermos‐mechanical model and raised an assumption that the SMPs were composed of hard frozen phase and soft active phase. The frozen phase locked (stored) the conformational rotation corresponding to the high temperature entropic deformation. On the contrary, the active phase allows localized free conformational motions. The volume fraction of the two phases was the function of temperature. By changing the ambient temperature, the ratio of the two phases could be adjusted and the required mechanical properties could be obtained. But this theory could not offer a good explanation for the time‐temperature equivalence of polymers.^[^ ^57^ ^]^ Furtherly, Qi et al.^[^ ^58^ ^]^ developed a three‐phase‐transition theory that combining the advantages of the viscoelastic theory and phase transition theory, giving a more accurate constitutive model for SMPs. SMPs are used to make actuators due to their reversible thermal actuations. Behl et al.^[^ ^59^ ^]^ prepared temperature memory polymer actuators (TMPAs) with a temperature‐memory effect for a long time. The actuators were able to undertake more than 250 cycles of thermal control actuation with the performance of the TMPAs rarely changed. Farhan et al.^[^ ^60^ ^]^ used SMPs to design a thermal response actuator with twisted shapes. With the change of the temperature, the actuator would be reversibly switched in continuous angles to indicate the temperature changes. Utilizing the strategies of structural design, many metamaterials with special functions are produced. Zhao et al.^[^ ^61^ ^]^ design lattice metamaterials with dual morphing modes. The structures were assembled by pre‐arranged soft phase and hard phase. At low temperature, the mechanical properties were decided by the whole zig‐zagged structures, and at high temperature, the thermal‐soften phase would symmetrically buckle under pressure and resulted in the second deformation mode (**Figure** **2**a). Jin et al.^[^ ^62^ ^]^ developed an origami robot with SMP networks. As Figure 2b shows, the bird‐like robot could be adjusted to various predesigned postures with the change of the temperature. Yuan et al.^[^ ^18^ ^]^ utilized a flexible elastomer and a relatively stiff polymer to fabricate new lattice architectures. As the temperature increased, the modulus of the amorphous polymers would decrease due to the glass phase transition, and then triggering a two‐stage pattern switching of the whole lattices (Figure 2c). Yuan et al. developed some pattern switches through predesign the layouts of the two components. Similarly, Chen et al.^[^ ^63^ ^]^ assigned different SMPs into conventional auxetic lattice configuration to gain the multilevel metamaterials, which could be used to embedded the wearable devices. In addition to the SMPs materials, some other special structures have also been developed, which achieved phase transition effect not from the materials but the structural configuration itself. Such as the interesting solid‐solid phase transformation effect in the specially designed microstructures.^[^ ^64^, ^65^, ^66^ ^]^ Wang et al.^[^ ^67^ ^]^ utilized the principle of jamming phase transition to fabricate a novel fabric (Figure 2d). When pressure was exerted on the boundaries of the chain mails, the internal units would interlock and change the properties of the structures.

#### 2.1.2 Strain Mismatch

Strain mismatch refers to the discontinuous changes of strain in medium, often occurring in architectures composed of two or more different kinds of materials. Due to the different mechanical properties, the uncoordinated strain of each part results in internal stress at the interface under the effect of environmental or load conditions, which furtherly lead to the bending or deformation of the structure. Based on this, metamaterials with active deformation and controllable response could be realized through subtle materials deployment. The study about strain mismatch could be traced back to the early 20th century. In 1925, the thermal deformation of a bi‐metal beam that composed of two kinds of materials with different thermal expansion coefficients was discussed by Timoshenko.^[^ ^68^ ^]^ The author obtained the deformation and buckling curvature, which laid the foundation for the study of strain mismatch. Later, some researchers studied the mechanical principle of strain mismatch in different conditions. Kim et al.^[^ ^69^ ^]^ derived the mechanical behavior model of a microcantilever with deposition of another material on it. In their calculation, the problem was considered as the strain mismatch of a double‐layer beam. In another application background, Xiao^[^ ^70^ ^]^ studied the strain mismatch problem of bilayer gel structures. In recent decades, most researchers mainly focus on how to utilize the effects of strain mismatch to provide the metamaterial with appropriate and controllable deformation. There are mainly two kinds of construction principle based on strain mismatch effect, namely, the strain mismatch phenomenon caused by temperature effect and the ones caused by swelling and deswelling effect.

**Figure 3:**
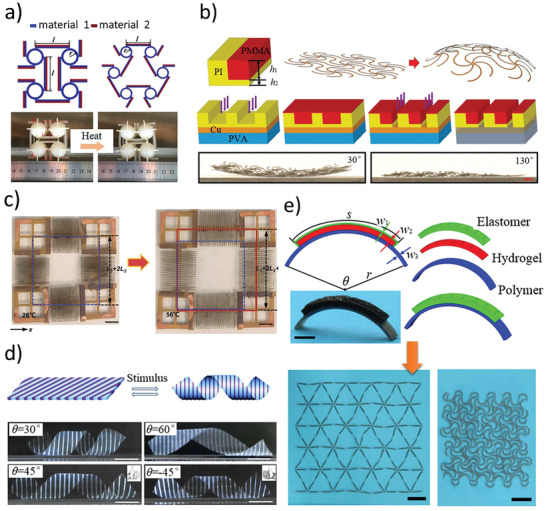
Metamaterials based on strain mismatch. a) NTE metamaterials that composed of different thermal expansion coefficient materials.^[^ ^11^ ^]^ Copyright, 2016, American Chemical Society. b) 3D adjustable thermal expansion deformation.^[^ ^73^ ^]^ Copyright 2019, Wiley‐VCH. c) Kirigami structures with positive expansion effects.^[^ ^74^ ^]^ Copyright 2021, Wiley‐VCH. d) Hydrogel‐driven helical structure.^[^ ^76^ ^]^ Copyright 2013, Nature Publishing Group. e) Soft mechanical metamaterials with negative swelling behavior.^[^ ^77^ ^]^ Copyright 2018, American Association for the Advancement of Science.

A typical design strategy is to combine the materials with different thermal expansion coefficients into composite beam. When the temperature increases, the side with high thermal expansion coefficient generate a larger strain, which makes the composite beam bend toward the side with low thermal expansion coefficient. Therefore, the strain mismatch principle is very suitable for realizing the transformation of dimensions. Ding et al.^[^ ^71^ ^]^ combined materials (glassy polymer and elastomer) with different thermal expansion coefficients to form a composite rod which was able to transform from a 1D rod to 3D structure. Tian et al.^[^ ^72^ ^]^ reported a Gaussian‐preserved shape‐morphing system with VO_2_ nano‐membrane and strip‐shaped Cr layer. When the system was stimulated by a force or thermal stimuli, it would transform from 2D flat shape into 3D rolled shape. Due to the fast response characteristics of VO_2_, the shortest response time of the system could reach 4.5 µs. In addition, unconstrained homogeneous materials generally yield uniformly expansions or contractions as the temperature rises or falls, which corresponds to a positive coefficient of thermal expansion. Once the concept of composite structural design was introduced, the structure with NTE property could be generated from the materials with positive coefficients of thermal expansion. Wu et al.^[^ ^11^ ^]^ utilized the deformation characteristics of anti‐chiral negative Poisson's ratio structure (**Figure** **3**a) to design and prepare AMMs. When the temperature rose, the structure contracted because of the composite ligaments’ bending. The innovation point of this work was that they developed the conventional 2D anti‐chiral metamaterials into 3D configuration, to achieve an overall NTE. Ni et al.^[^ ^73^ ^]^ achieved the control of the thermal expansion tensors of the structure in combination with the serpentine lattices of six ligaments, and transformed the 2D architecture to a 3D curved surface by adjusting the combination forms between different materials, as is shown in Figure 3b. Different from the construction principle that combining diverse materials into composites, Guo et al.^[^ ^74^ ^]^ designed metamaterials inspired by the kirigami microstructure, achieving giant tunable positive and NTE. The active metamaterials could effectively transform the thermal mismatch into colossal expansion or contraction force, as shown in Figure 3c. Also based on these kirigami metamaterials, Yu et al.^[^ ^75^ ^]^ furtherly performed mechanical designs, theoretical predictions, and experimental demonstrations to complemented this work.

Similar to the mechanical construction principle based on thermal strain mismatch effect, the utilization of swelling mismatch effect is another design strategy to AMMs. Water‐responsive hydrogels are a kind of typical hydrophilic polymer materials, which exhibits obvious deformations when absorbing or losing water.^[^ ^78^, ^79^ ^]^ According to this property, many researchers have developed AMMs with special deformation modes. Wu et al.^[^ ^76^ ^]^ alternately arranged two kinds of hydrogel stripes with different shrinkage and elastic modulus at the certain angles, to prepared a planar sheet structure. Due to the internal stresses in the NaCl solution, the products were able to realize self‐driven spiral bending deformation in accordance with the pre‐set mode (Figure 3d). Mao et al.^[^ ^80^ ^]^ demonstrated a periodical macrostructure that was able to realize self‐folding and unfolding process by combining hydrogels with the SMPs. The hydraulic swelling force of hydrogels resulted in morphing, and the SMPs would be used to regulated the rate of morphing. Though stiffness and deformation of the hydrogels are relatively easy to be controlled, the ductility and flexibility of the hydrogels are weak. And these two problems are challenging in the scientific research of hydrogels.^[^ ^79^ ^]^ Zhang et al.^[^ ^77^ ^]^ demonstrated a reversible component design: employing the polymers as the substrate, the perforated elastomer as the cover layer, and the hydrogels as fillers, as is shown in Figure 3e. The strip shaped units were assembled into a negative swelling networks with hexagonal chiral honeycomb configuration. And the experiment showed that the metamaterials could reach a large negative swelling ratio up to 98%. With a similar design strategy, Chen et al.^[^ ^81^ ^]^ applied the swelling strain mismatch to origami structures, and developed AMMs with positive, negative, and zero swelling behavior. But in this work, the layer is thin. When the metamaterials undertook multiple driving deformation, the local deformation tended to be imbalanced due to the thin, delicate encapsulation, and leading to the uniformity of overall structural deformation. In addition, the reaction time of AMMs based on swelling strain mismatch design is relatively long. Therefore, this design scheme is not suitable for cases that requiring rapid response.

#### 2.1.3 Instability

Traditionally, the design of engineering structures aims to avoid the instabilities to ensure safety. But in recent decades, researchers turned to take advantage of various instabilities to design novel structures and metamaterials. The mechanical construction based on instability mainly includes two aspects: 1) The instabilities in microstructured materials, which is of the micro scale, and 2) the structural instabilities, which is of a macro scale.

Petryk^[^ ^82^ ^]^ systematically summarized the material instabilities in elastic materials and plastic solids, which built a theoretical framework for the studies and metamaterials design based on the microstructured materials instabilities. Kochmann et al.^[^ ^83^ ^]^ regarded the composite materials as the combination of several phases. The homogeneous linear elastic phases had positive‐definite moduli, while the phases with nonpositive‐definite elastic moduli had negative stiffness. These constituents were temporarily stable due to their mutual constraints. Nonconvex potential energy landscape was the key to understand the material instabilities. According to the principle of minimum potential energy, microstructured materials tended to be arranged with the energy‐minimizing sequence. The materials with non‐convex energetic potentials would skip the uniform deformation and rapidly jump to the position with low potential energy, which exhibiting macroscopic instabilities including phase transformations, domain patterning, strain localization, etc. Consequently, the core of the material‐instability‐based metamaterial design is to controlling the number of the minimum potential energy points of the materials, and the time when the minimum potential energy points appear during the loading process. Li et al.^[^ ^84^ ^]^ focused on the elastic instabilities in common soft materials, discussing the progress in soft porous materials, heterogeneous multiphase and fiber composites. They also explained the effect of post‐buckling in the various mechanisms of instabilities. Viard et al.^[^ ^85^ ^]^ studied the propagations of instability in different 2D lattices that were respectively bending‐dominated and stretch‐dominated. Through the experiment, they proved that the initiation and propagation of instabilities could be controlled by the microstructures, which provided the reference for more material‐instability‐based metamaterials.

**Figure 4:**
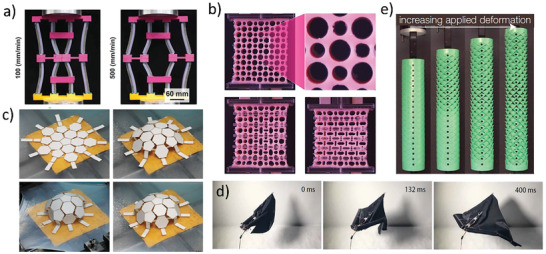
Metamaterials based on mechanical instability. a) Buckling deformation of double beam sensitive to strain rate.^[^ ^86^ ^]^ Copyright 2020, American Association for the Advancement of Science. b) Porous metamaterials with tunable mechanical behaviors.^[^ ^90^ ^]^ Copyright 2014, American Physical Society. c) 2D to 3D transformation process of soccer‐shaped active metamaterials.^[^ ^101^ ^]^ Copyright 2016, Wiley‐VCH. d) Bionic self‐folding flap robot based on mechanical instability principle.^[^ ^104^ ^]^ Copyright 2020, American Association for the Advancement of Science. e) The crawling of the python like robot with kirigami skin.^[^ ^31^ ^]^ Copyright 2019, National Academy of Sciences.

Structural instability refers to the phenomena that the structure loses its stable state when the applied load increases to a threshold value. If the load continues to increase by a small increment, the deformation will rapidly increase and accelerate the failure of the structure. Common elastic instabilities include, for example, structural curving, buckling, twisting, wrinkling, folding, and indentation, etc. These large deformations and rotations result in large pattern transformations and mode switching of the structures, which could be widely utilized for the design of structural‐instability‐based metamaterials. Janbaz et al.^[^ ^86^ ^]^ bonded two kinds of materials with respectively hyperelastic and viscoelastic properties to prepare composite beams, which showed different deformation modes with the change of strain rates. Based on this, they used a predictable analytical model to explain the instability of the beams and constructed the complex strain rate‐dependent systems (**Figure** **4**a). Among previous works, structural instability has played an important role in guiding the design of various multi‐stable structures.^[^ ^87^ ^]^ Tao et al.^[^ ^39^ ^]^ combined the two kinds of SMPs in a corrugated configuration to obtain the additional thermal response effect. When the ambient temperature increased, the relatively weak SMP layers buckled under the compression load while the strong layers maintained their shapes, resulting in regular multi stable states. Hence the deformations and recovery modes of the structures could be switched and pre‐programmed. Based on structural instabilities, various porous metamaterials have been developed,^[^ ^88^, ^89^, ^90^, ^91^, ^92^ ^]^ as shown in Figure 4b. The shapes and sizes of the micro‐holes were controlled by geometric constraints or loadings on the laterals, which had a huge impact on the materials. Therefore, multiple characteristics of the materials, such as, the failure modes, mechanical properties, propagating elastic waves, and the discontinuous buckling could be adjusted flexibly. Besides, thin elastic shells show significant unstable post‐buckling stage with the continuously increasing external load. Marthelot et al.^[^ ^93^ ^]^ discussed the instability process of a pneumatic elastic thin shell with pre‐designed patterned surface. Due to the constrained buckling design, the structure would gradually lose the state of stability as the pressure decreased, and reticulated networks of sharp ridges emerged on the surface of the shell. Snap‐through is a common design strategy based on structural instability,^[^ ^31^, ^94^, ^95^, ^96^, ^97^ ^]^ and it was usually used to produce mechanical metamaterials with multiple stable states. Most snap‐through metamaterials are constructed using the instability of elastic beams. Fu et al.^[^ ^94^ ^]^ attempted a novel approach by wrapping the granular particles with membrane to generated designable instabilities. These metamaterials with typical zig‐zag force‐displacement curves and tunable peak forces also showed good energy absorption capacity. Based on structural instabilities, pre‐strain design strategy^[^ ^98^, ^99^, ^100^, ^101^, ^102^, ^103^ ^]^ inspired a large class of metamaterials. Yan et al.^[^ ^101^ ^]^ designed an autonomic 3D structure using the mechanism of buckling effect of the 2D precursor. The spatial variation of thickness of the 2D structure was preset and attached to the substrate. When the pre‐stretched elastomer substrate was released, the structure could flexibly transform from 2D configuration to expected 3D configuration (Figure 4c). Based on this idea, Yan^[^ ^102^ ^]^ and coworkers extended this concept to multilayer and multi‐material mesostructures and furtherly designed 3D near‐field communication devices.

In nature, buckling instabilities are widespread and mostly appear in the form of membrane structures. Such as the growth processes of plant leaves,^[^ ^105^, ^106^, ^107^ ^]^ the ladybird beetle flapping wings,^[^ ^104^ ^]^ the predatory action of flytrap,^[^ ^108^ ^]^ the skin of snakes,^[^ ^31^, ^97^ ^]^ etc. Those examples provide inspiration for structural design, especially the design of soft robots. Huang et al.^[^ ^106^ ^]^ studied the physical process of plant leaves growth and revealed that the morphological transition process was driven by the instability effect caused by the differential growth of each part of the tissues. Baek et al.^[^ ^104^ ^]^ imitated the structure of ladybird beetle wings to design a soft flying robot. When it folded, the elastic energy was stored on the deformed facet and realized self‐locking; and this energy would release to make the robot rapidly deploy within 116 ms. This self‐locking and self‐deployment ability provide an extremely high load‐bearing capacity in flight (Figure 4d). Inspired by the bistablility and developability of the Venus flytrap leaves, Kim et al.^[^ ^108^ ^]^ presented a soft robot with the ability to rapid and large morphing movements. Inspired by the snake crawling and kirigami, Rafsanjani et al.^[^ ^31^, ^97^ ^]^ designed some python‐like robots with kirigami shells. When the pneumatic cylindrical shell stretched (Figure 4e), the flat surface would transform into a 3D‐textured surface and gripping the ground to generate friction. The phase transition characteristics could be controlled by presetting the geometry of the cut and curvature of the kirigami skins.

### 2.2 Simulation and Data‐Driven Methods

In addition to the mechanical construction principle and experimental testing strategies for the design of metamaterials, with the development of computer science, computer aided design and computer aided engineering have played more and more important roles in the development of metamaterials. At the beginning of the metamaterial design, numerical simulation could effectively predict the feasibility of the design scheme. During this design process, data‐driven artificial intelligence and other methods could be used to shorten the design cycle by training the computational model with experimental data, and thereby improve efficiency. When experiments were completed, the experimental results could be checked by simulation. Therefore, simulation and data‐driven methods are becoming more and more indispensable in the design of metamaterials.

#### 2.2.1 Structural Optimization

Structural optimization generally includes size optimization, shape optimization, topography optimization and topology optimization. Among them, topology optimization implies arranging the distribution of the materials to obtain the desired performance of the structure within the specified design domain.^[^ ^109^ ^]^ The main idea of the topology optimization method in mechanics is to express various mechanics indexes of the structure as a function related to the material distribution, and establish optimization algorithms with constraints to find the optimal solution, and finally optimize a specific performance of the material. Such as ultra‐lightweight and high‐strength advanced structures.^[^ ^110^ ^]^ By now, the topology method has developed tremendously and has been used in the design of the AMMs. Sigmund et al.^[^ ^111^ ^]^ utilized numerical topology method to create metamaterials with positive, zero and NTE coefficients. Here, the topology method guided the distribution of the matrix phase and the void phase, and thus minimizing the objective function. Wang et al.^[^ ^112^ ^]^ established a bi‐objective optimization to model to design active metamaterials with directional thermal expansion functions and high stiffness. Furthermore, Wang et al.^[^ ^113^ ^]^ fabricated multifunctional metamaterials. They evaluate the effective properties of the microstructures with numerical homogenization method and evolve the boundaries with the level‐set optimization method. Geiss et al.^[^ ^114^ ^]^ proposed a density topology optimization method for 4D printed structures. Clausen et al.^[^ ^115^ ^]^ prepared a series of architectures with programmable Poisson's ratio under large deformations. The topology optimized nonlinear configuration turned out to be more stable than the traditional linear configuration, and the Poisson's ratio of the nonlinear configuration kept constant as the strain changed from 0 to 0.2.

#### 2.2.2 Machine Learning

Traditionally, the emergence of new mechanical metamaterials is generated through a large number of mechanical experiments and numerical simulations, which often depend on the experience of the designers or through trial‐and‐error. Therefore, previous methods are complicated in experiments, long in the research cycle, and highly cost in development. These disadvantages are exactly what machine learning is good at solving. In recent years, introducing machine learning into the design of metamaterials has become a new research hotspot. In general, machine learning refers to the process of instructing the computer to obtain an appropriate model based on existing data. The existing experimental and simulation data could be organized as the results database. Then they selected proper algorithm model to establish the relation of the input parameters (such as, types of materials, micro‐architectures, etc.) and output parameters (such as, stiffness, flexibility, compressibility, etc.). After being trained by large amounts of data, the obtained model can predict the properties of metamaterials or tailor the micro‐architectures for metamaterials according to external conditions. So the current challenging problems in the design of AMMs are likely to be solved through machine learning. Hamel et al.^[^ ^116^ ^]^ reported a kind of active composite structures based on machine learning. Combining finite element method and evolutionary algorithm, the target shape shifting responses of the composites could be accurately achieved. Wu et al.^[^ ^117^ ^]^ introduced evolutionary algorithm into additive manufacturing process. The desired voxel distribution would be obtained after the pre‐design evolutionary algorithm iterations terminated, and the motions and curvature distributions under magnetic actuation could be programmed. Machine learning uses computers to mine the potential value from data and learn the objectives’ laws and characteristics. But it should be noted that whether a machine learning model keeps accuracy outside the dataset is always posteriori. So the independent variables should be kept in the training data space to ensure the correctness of the model.

## 3 Active Mechanical Metamaterials Responsive for Various Stimulus Fields

### 3.1 Thermal‐Responsive Active Mechanical Metamaterials

Thermal energy is one of the most common forms of energy in nature, and thermal‐actuation approaches have been used by people in the design of active materials for a long time. The basic principle of the thermal‐actuation method is to change the properties or shapes of the thermal‐responsive materials by controlling the heat exchange. In a broad sense, the approaches that employing thermo‐chemical reactions, photo‐thermal effects, electro‐thermal effects, and magneto‐thermal effects all could be classified as thermal‐actuation methods. Therefore, the thermal‐actuation method is essentially the most widely used actuation method in AMMs. Common thermal‐responsive materials used in AMMs include SMPs,^[^ ^19^, ^38^, ^118^, ^119^, ^120^, ^121^, ^122^ ^]^ SMAs (shape memory alloys),^[^ ^123^, ^124^, ^125^, ^126^, ^127^, ^128^ ^]^ thermal‐responsive LCEs,^[^ ^32^, ^33^, ^35^, ^129^, ^130^, ^131^ ^]^ and thermal‐responsive hydrogels.^[^ ^132^, ^133^, ^134^, ^135^, ^136^ ^]^ Either directly heating the metamaterials themselves or transferring heat to them by adjusting the temperature of the external environment, the shape, mechanical properties, and functions of the metamaterials would be adjusted effectively.

**Figure 5:**
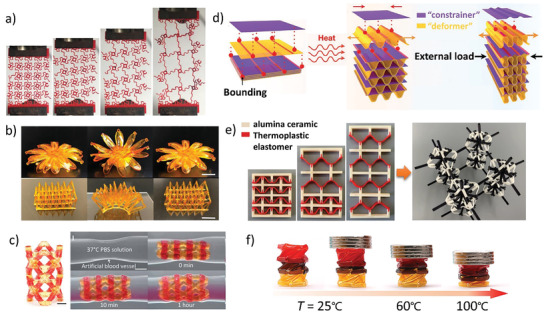
Thermal‐responsive active metamaterials. a) Uniaxial stretching diagram of 4D printing auxetic metamaterials.^[^ ^121^ ^]^ Copyright 2020, Wiley‐VCH. b) The actuation and recovery of a flower and bi‐stable lattices.^[^ ^35^ ^]^ Copyright 2021, Elsevier. c) Display of the drug release function of SMP‐hydrogel stent.^[^ ^136^ ^]^ Copyright 2021, American Association for the Advancement of Science. d) Composition and deformation principle of super‐elastic thermal‐responsive metamaterials.^[^ ^138^ ^]^ Copyright 2020, Wiley‐VCH. e) Bi‐material multi‐stable metamaterials and the three‐dimension configuration in a stable state.^[^ ^20^ ^]^ Copyright 2019, Elsevier. f) The assembly deforms in sequence during the heating process to realize multi‐shape response functions.^[^ ^37^ ^]^ Copyright 2020, Elsevier.

SMPs are the most commonly used thermal‐responsive materials, and the phase transition principle has been depicted in Section 2.1.2. Generally, a typical thermal‐sensitive shape memory period includes the following steps:^[^ ^137^ ^]^ 1) Fabricating the materials into an original shape; 2) heating (*T > T*_trans_) and loading to shape the materials; 3) cooling down (*T < T*_trans_) and unloading the force, making the materials retain the temporary shape; 4) reheating (*T > T*_trans_) to make the materials recover to the original shape. *T*_trans_ represents the switching transition temperature. Combined with the typical configurations of mechanical metamaterials such as lattices, auxetic structures and origami structures, a large number of thermal‐responsive AMMs have been developed. Yang et al.^[^ ^19^ ^]^ developed lightweight lattices AMMs whose stiffness would significantly change with the temperature changed from 30 to 90 °C. The structures were able to significantly absorb the shock energy when subjected to impact loading, as well as restore their original shape and stiffness even after large deformation. Xin et al.^[^ ^121^ ^]^ made auxetic metamaterials with 4D printing technology and studied the relationship between the mechanical properties and the deformation. Due to the chiral microstructures, the structures would realize large deformations, programmable mechanical properties, tunable and reconfigurable configuration (**Figure** **5**a). Lei et al.^[^ ^119^ ^]^ and Wang et al.^[^ ^38^ ^]^ also developed metamaterials that were responsive to the thermal environment based on chiral architectures. Ding et al.^[^ ^118^ ^]^ made some simple prepared thermal‐responsive active metamaterials. The structures could be programmed from the plane state into a variety of shapes such as tube, rod, shell, sector, etc. under thermal excitation. Based on triangular cylindrical origami (TCO) metamaterial, Tao et al.^[^ ^38^ ^]^ (SMPs) utilized another strategy and also obtained the tunable mechanical properties. They found that when heated to the same temperature, the TCO elements with different relative angles of the upper and lower polygons tended to have different stress–strain curves. According to this principle, different TCO units were combined to configured programmable compression‐twist structures. Apart from SMPs, some SMAs responding to thermal stimuli were used as components in AMMs. Zhao et al.^[^ ^127^ ^]^ broke through the limitations of the traditional design and developed structures with both large actuation deformations and high load‐bearing capacities. The 3D printing frame provided a large deformation capacity, while the SMA spring inside provided adjustable stiffness. By changing the assembly orders and load directions of the units, the functions of the structures were greatly expanded. It should be noted that the structure could also be driven by electric heating. Yang et al.^[^ ^128^ ^]^ utilized the concept of “bit” in computer science and designed a small circular kirigami unit. The thermal expansion coefficients of each side of the cubic frame structures were able to switch between 8 modes in the three directions of x, y, and z, so the whole structure was highly programmable. LCEs is another typical type of common used thermal‐responsive materials for AMMs. LCEs combine the elastic properties of elastomers and the anisotropy of liquid crystals, exhibiting large and reversible deformations when subjected to the stimuli. Yuan et al.^[^ ^129^ ^]^ embedded the LCEs strips into the multi‐layered structures through 3D printing. When stimulated by Joule heat from the conductive wires, the LCE layer would bend and leading to a two‐way shape changing behaviors of the actuators. Moreover, Minori et al.^[^ ^130^ ^]^ adopted a more complicated lamination strategy to prepare deployable machines. Figure 5b shows some thermal responsive structures developed by Peng et al.^[^ ^35^ ^]^ with elastomers as the matrix and LCE fibers as the actuation components. Though not the best thermal‐responsive materials, some hydrogels are still used for thermal actuations due to their unique properties in the water environments. Thermal‐responsive hydrogels could be classified into three main categories, including hydrogels with polymer phase separation, with glassy‐like transition, and fusible links.^[^ ^79^ ^]^ Through multi‐material 3D printing technology, Ge et al.^[^ ^136^ ^]^ fabricated multi‐material structures, such as, combining acrylamide‐PEGDA (AP) hydrogel based components with elastomers, or combining three components of rigid materials, AP hydrogels, and elastomers. They also embedded some rigid polymers as skeleton into the hydrogel matrix to achieve the purpose of toughening the structure. As shown in Figure 5c, they prepared an SMP‐hydrogel stent to deliver drugs inside the liquid environment of human body. The stent could release drugs slowly under the regulation of human body temperature.

Not limited to the above mentioned three types of thermal‐responsive materials, many new thermal‐responsive AMMs based on the strategy of assembly have been developed. Wu et al.^[^ ^138^ ^]^ used the thermal‐responsive polyester films as “constrainers” and thermal‐steady polyimide films as “deformers.” By stacking the films layer by layer, Wu et al. prepared the metamaterials with extensible dimensions, recoverable deformation, and self‐adaptive stiffness at specific temperatures (Figure 5d). This multi‐material strategy has increasingly become more and more popular for mechanical metamaterials design. Hierarchical structures are another representative metamaterials design method. A lot of thermal‐responsive active hierarchical metamaterials emerged recently, whose mechanical properties were highly controllable and programmable. Yang et al.^[^ ^20^ ^]^ extended the concept of multi‐stable structure design from 1D to 3D (Figure 5e), which made the number of the stable‐state greatly increased and the thermal expansion coefficient highly adjustable. Boatti et al.^[^ ^139^ ^]^ designed a mechanical metamaterial with tunable thermal expansion coefficients. They arranged the softness and hardness of the origami panels and creases at specific positions (soft parts were single layers of paper, and hard parts were layers with polyethylene film attached on). As the temperature rose, the different designed samples showed positive, negative, and zero thermal expansion coefficients. Fang et al.^[^ ^37^ ^]^ employed the concept of modular 4D printing to prepare structures for each module with different glass transition temperatures. The deformation of the entire structure as the temperature change could be arbitrarily configured (Figure 5f).

### 3.2 Chemical‐Responsive Active Mechanical Metamaterials

In addition to the thermal‐response active metamaterials, various chemical methods are applied to the design of active metamaterials. Most chemical‐responsive materials function relying on the liquid environment, such as water, acid, organic, or ionic solvent.^[^ ^140^ ^]^ Among them, hydrogels have received extensive attentions due to their good hydrophilic properties. Hydrogels are a kind of soft materials composed of cross‐linked hydrophilic polymer networks, and the representative characteristics are their strong capabilities to absorb and discharge water. When stimulated by external chemicals such as salinity, pH, temperature, or humidity, hydrogels would spontaneously change their shapes.

**Figure 6:**
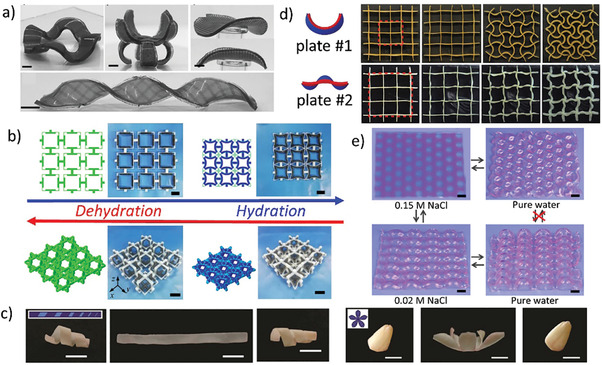
Chemical‐response active metamaterials. a) Samples with patterns on both sides and the deformed shapes.^[^ ^143^ ^]^ Copyright 2017, Wiley‐VCH. b) Initial state and the hydration state of the 2D and 3D auxetic metamaterials.^[^ ^145^ ^]^ Copyright 2020, Elsevier. c) Shape memory demonstration of the wax‐based polymers.^[^ ^148^ ^]^ Copyright 2017, Wiley‐VCH. d) The swelling process of square lattices with different unit configurations (plate #1, plate #2).^[^ ^149^ ^]^ Copyright 2016, Wiley‐VCH. e) Photolithographic patterning of gels and swelling‐induced cooperative deformation.^[^ ^150^ ^]^ Copyright 2017, American Association for the Advancement of Science.

The potential for chemical‐response hydrogels provides abundant ideas for the design of AMMs.^[^ ^141^, ^142^, ^143^, ^144^, ^145^, ^146^, ^147^ ^]^ Palleau et al.^[^ ^144^ ^]^ presented an ionoprinting, patterning, and actuation method. The patterned hydrogels exhibited programmability and flexible spatial deformability. Peng et al.^[^ ^142^ ^]^ presented a facile and versatile ion dip‐dyeing or ion transfer printing technology. They printed simple patterns on the 1D, 2D, and 3D hydrogel structures, which would change into more complex structures with higher dimensions. After that, they^[^ ^143^ ^]^ printed batch complex patterns on the surfaces of large‐scale hydrogel samples, so that the deformation rates and degrees of the hydrogels could be accurately programmed by printing time and the patterns (**Figure** **6**a). Hao et al.^[^ ^141^ ^]^ proposed a kirigami design and control strategy of deformation structure, by which they fabricated porous composite hydrogel sheets with programmable and multi‐stable 3D configurations. The active and high‐swelling hydrogel strips were embedded in the stiff frames, and the buckling direction of each strip was controlled by a pressure step. Thus the composite hydrogels were capable of switching different stable states in water. Wei et al.^[^ ^145^ ^]^ designed self‐driven auxetic metamaterials with negative hydration expansion function (Figure 6b) and studied the deformation mechanism and several macro deformation modes of these metamaterials. Despite these advances, when compared with their natural analogs, the shape‐deformation system of hydrogels is still at an early stage of development.

From the above works, the existing hydrogel‐based structures are weak and fragile for many applications under harsh environments. Compared with hydrogel metamaterials, active metamaterials based on SMPs and elastomers have stronger mechanical properties, and suitable for other application backgrounds. Huang et al.^[^ ^148^ ^]^ prepared the wax‐based SMPs. Hydrophobic lauryl acrylate (LA) was used as the monomer, and 1,6‐hexanediol diacrylate as the cross‐linker. The products would deform at high temperature after wax melting, and then fix into a temporary shape when it was cooled down to room temperature. And this kind of material possessed the characteristics of programmable deformation and reconfigurable shapes (Figure 6c). Wang et al.^[^ ^151^ ^]^ proposed active metamaterials with an obvious water‐driven shape memory effect. The immersion experiments showed that the chemical and physical expansion effects have a significant impact on the water‐induced shape memory process. The auxetic metamaterial proposed by Liu et al.^[^ ^149^ ^]^ consisted of series of double‐layer plates. The two layers of the plate had different swelling properties. When absorbing solvent, the laminated plates tended to bend out of the plane, which resulted in a negative swelling effect and a reduction in the distance between the two ends, as shown in Figure 6d.

Another kind of chemical‐responsive active metamaterials is realized by the addition of metal cations. The metal cations inside are not only used to regulate the deformation but also improve the mechanical properties of the active metamaterials. Fe^3+^ ions are strong cross‐linker which leads to the formation of the second ionically cross‐linked network of the gel materials. Therefore, when Fe^3+^ ions were diffused into the Ca‐alginate/PAAm (polyacrylamide) tough gel matrix, the highly stretchable gels would become stiffer and harder to stretch. And this phenomenon was described as “freeze” by Li et al.^[^ ^21^ ^]^ They patterned the Fe^3+^ ion into gels to introduce anisotropy so that the deformation of the metamaterials could be accurately controlled. Based on similar principles, Zhou et al.^[^ ^22^ ^]^ proposed a mechanical‐chemical controlled origami. The bending process of origami was based on the stiffness mismatch of locking components (Fe^3+^) and unlocking components (Ca^2+^) in the pre‐stretched tough gel. Moreover, the rotation mode could be realized by introducing chirality into the kirigami configuration for programmable deformations. Wang et al.^[^ ^150^ ^]^ dispersed non‐swelling gels in high‐swelling gels to prepare composite gel sheets. The synergistic deformation of 2D periodic pattern hydrogel sheets was adjusted by the concentration of Na^+^ ions in the solution. In solution, the structure was spontaneously deformed into a 3D alternating concave‐convex configuration due to the strain mismatch caused by the difference in expansion ratio, as shown in Figure 6e. These methods provide a novel and simple way to create supramolecular structures based on the tough gel and expected to be widely used in biomedical devices and soft robots.

pH‐responsive is also a popular method for active metamaterials. Due to the specific pH requirements of many specific environments, the pH‐response characteristics are widely used in sensors or biomedicine fields.^[^ ^152^ ^]^ The intermolecular hydrogen bonds in gels could be broken or formed by the protons in acidic solution, resulting in reversible sol–gel transition and a series of changes of mechanical properties such as yield stress and viscoelasticity.^[^ ^153^ ^]^ This is a typical mechanism of pH‐sensitive gels. Hong et al.^[^ ^154^ ^]^ designed a calcium alginate gel microsphere structure capable of embedding platinum compounds, and Shim et al.^[^ ^155^ ^]^ designed a tunable hydrogel bilayer structure for microcapsules. Both of them carried out in vitro drug release experiments by simulating the pH environment of the gastrointestinal fluid. The results showed that the change of pH value led to the gradual decomposition of the microsphere carrier, or the highly reversible transformation between planar microparticles and the microcapsules. Bassik et al.^[^ ^156^ ^]^ prepared pH‐responsive metamaterials from composite hydrogels with the best swelling response. The bilayer structure was triggered by the changes in pH and ionic strength, to transform from 2D configuration to 3D configuration. This method provides a simple way to design and manufacture actuator hinges entirely composed of polymers.

### 3.3 Light‐Responsive Active Mechanical Metamaterials

The light‐responsive properties are achieved by mixing light‐responsive functional groups as molecular switches or photo‐thermal conversion agents in polymer networks. Therefore, the light response can be roughly classified into photochemical and photo‐thermal.^[^ ^157^ ^]^ Deformations of the light‐responsive components are stimulated by external light sources, so that the structures are triggered to deform or move on a macro scale.

**Figure 7:**
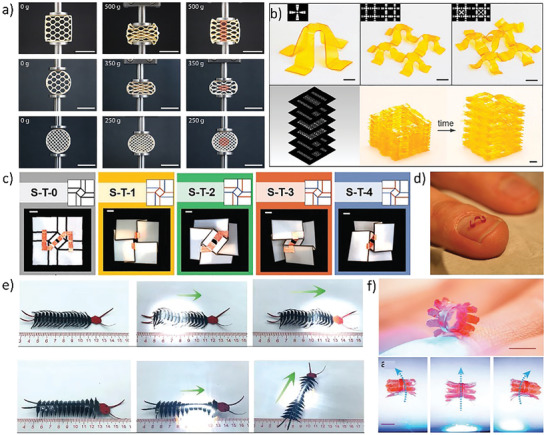
Light‐responsive active metamaterials. a) Comparison of compression results of light‐response metamaterials with and without light source stimulation on the local region.^[^ ^161^ ^]^ Copyright 2020, Wiley‐VCH. b) Origami metamaterials with different numbers of pop‐up units and the schematic of unfolding effect under light stimulation.^[^ ^162^ ^]^ Copyright 2020, American Physical Society. c) Five typical modes of square twist origami antenna engagement.^[^ ^24^ ^]^ Copyright 2020, Wiley‐VCH. d) Light‐controlled microrobot.^[^ ^157^ ^]^ Copyright 2017, Wiley‐VCH. e) SLiRs (somatosensory light‐driven robots) centipede shows two kinds of movements: Waving forward and rotate turning.^[^ ^53^ ^]^ Copyright 2020, Wiley‐VCH. f) Flexible rolling of light‐responsive ratchet robot.^[^ ^50^ ^]^ Copyright 2020, Wiley‐VCH.

The photochemical effect is based on the photoreaction of the light‐responsive functional groups, for example, photoisomerization and photodimerization.^[^ ^158^ ^]^ When exposed to light with a special wavelength and frequency, the photochemically reactive molecules will form photoreversible covalent crosslinking points in polymers, then the reversible photochemical reaction is achieved by the crosslinking and de‐crosslinking process of the polymer network.^[^ ^159^ ^]^ As a unique advantage, this actuation method can be independent of any temperature effect. For instance, cinnamic groups (CA) were typical kinds of photochemically reactive polymers. When exposed to the irradiation of UV (ultraviolet) light of *λ*_ _> 260 mm, the polymer could be stretched into different shapes and cured. And these shapes could maintain stability even when heated to 50 °C. When irradiated with UV light of *λ*_ _< 260 mm, the material would return to its original shape.^[^ ^160^ ^]^ Similarly, photoreactive cinnamates had the property of reversible cycloaddition reaction when illuminated with wavelength >300 nm, which lead to a significant change in Young's modulus. Müller et al.^[^ ^161^ ^]^ took advantage of this to graft photoreactive cinnamates onto the surface of CNC (cellulose nanocrystals) and easily prepared some kinds of single‐material honeycomb mechanical metamaterials by direct ink writing (DIW). When irradiated by light, Young's modulus of the specific area (marked in red) was increased because of the generation of a second polymer network, as shown in **Figure** **7**a. In this way, the mechanical properties of the overall structure could be tailored.

The photo‐thermal effect is based on the photo‐thermal conversion agents which absorb light and convert it into thermal energy, thus the process is more simple when compared with the photochemical effect. And in essence, the photo‐thermal effect still belongs to the category of thermal response, so that similar design ideas could be used when designing metamaterial configurations. Based on the above discussion, the physical and mechanical properties of metamaterials will depend on both the characteristics of the light‐responsive materials and the architectures of the metamaterials. So that the photo‐responsive active metamaterials possess better and more tunable mechanical performance than traditional light‐responsive materials. Based on the volume shrinkage effect of polymer resin during photopolymerization, Zhao et al.^[^ ^163^ ^]^ developed a 3D deployable origami. They added a non‐uniform distributed light absorber to the polymer resin, so the layers of the materials would shrink unevenly along the thickness direction under the light. Based on that, the deformation of the origami structure would be adjusted by presetting the spatial grayscale and controlling the light time. Furthermore, Zhang and coworkers^[^ ^162^ ^]^ designed a mechanically robust and rapid shape‐shifting 4D printing material system (Figure 7b). First, the volatile substances in the polymer volatilized when heated by light, driving the deformation of the structure; the second step was the light curing process, which completely cured nonvolatile residual cross‐linkers and photo‐initiators in the polymer networks and making the structure robust. It was worth mentioning that this work was the first combination of grayscale 3D printing and volatilization to achieve 4D printing. Wang et al.^[^ ^24^ ^]^ studied the classic square‐twist origami structures with multi‐stable deformation modes (Figure 7c) and analyzed in detail the mechanics of its deformation when exposed to light. In this work, the light curing resin panels of the origami were photo‐thermal responsive. In addition, they adhered conductive copper patterns inside the origami and designed a series of frequency reconfigurable and programmable antennas, which were able to realize five more different working modes and programmable frequency reconstruction. Zhang et al.^[^ ^164^ ^]^ assembled polymer sheets and photosensitive hinges (made of pre‐strained polystyrene) to design a series of metamaterials. The hinges would shrink when exposed to light, and the folding direction and folding angle of the origami structure were guided by the preset thickness and width of the hinges. They developed multiple structures like self‐folding cylinders, spirals, and pyramids with zero Gaussian curvature.

Owing to the advantages of remote control, accurate and quick response, light‐responsive AMMs have many applications in the field of soft robotics, Zeng et al.^[^ ^157^ ^]^ utilized monolithic LCE film to design a microrobot by imitating the crawling movements of caterpillars (Figure 7d). The robot would be activated under a medium intensity light and was able to work on the blazed grating, human skin, etc. Wang et al.^[^ ^43^ ^]^ used rigid plastic rods as pressing rods and artificial muscle fibers as tension cables to construct a tensegrity robot. This structure was similar to the musculoskeletal structure of animals. Among them, the artificial muscles were composed of LCEs and carbon nanotubes (CNTs) composite, which would produce large and reversible deformation, thus allowing the robot to move driven and navigated by light. Wang et al.^[^ ^53^ ^]^ designed thin‐film SLiRs with kirigami configuration. The active materials were laminates of photo‐thermal response materials, conductive materials, and piezoelectric materials. As shown in Figure 7e, the centipede robot moves forward and turns under the navigation of the external light source. Different from the common designs of the photo‐sensitive materials simply as strip‐like or wire‐connection structures with limited freedom of movement, Cheng et al.^[^ ^50^ ^]^ employed kirigami architecture to design an interesting rolling robot. They adopted the design of the metamaterial similar to a ratchet wheel, to make the robot roll and climb slopes with high efficiency (Figure 7f).

### 3.4 Electro‐Responsive Active Mechanical Metamaterials

Electric driving methods have important applications in AMMs. They could be generally divided into two categories in principle. The first type is to utilize the thermal energy generated by current in the conductors to actuate mechanical deformation, which still belongs to the thermally driven method in essence. The representative ones are the SMPs and SMAs based on the electro‐thermal effect. Another type of electro‐responsive metamaterials is not actuated electro‐thermally but responds to external electrical stimulation through some physical or chemical reactions. Such as electrochemical response materials, dielectric elastomers of electroactive polymers, and ionic polymer‐metal composite materials. The review of Levine et al.^[^ ^165^ ^]^ has made a comprehensive summary of these materials, and they mainly focus on the stiffness change induced by electric field.

**Figure 8:**
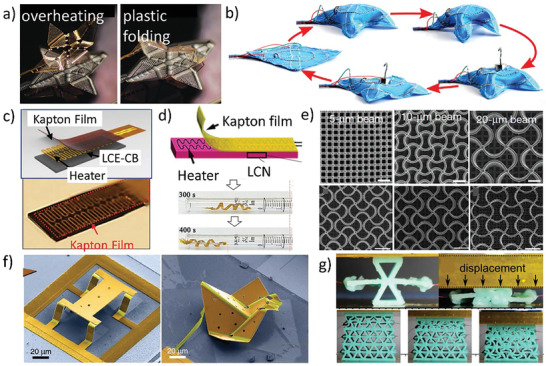
Electro‐responsive active metamaterials. a) The reprogramming process of the origami pattern by overheating.^[^ ^167^ ^]^ Copyright 2020, Wiley‐VCH. b) The transformation modes of the robot fabric, and the display of its bearing capacity.^[^ ^168^ ^]^ Copyright 2020, National Academy of Sciences. c) Reproduced with permission.^[^ ^169^ ^]^ Copyright 2018, Wiley‐VCH. d) Reproduced with permission.^[^ ^170^ ^]^ Copyright 2019, Wiley‐VCH, are schematics of the internal structures of laminated electro‐thermal response metamaterials. e) The lithiation process of auxetic lattices with different beam sizes.^[^ ^171^ ^]^ Copyright 2019, Nature Publishing Group. f) Micrometer‐scale origami quadruped robot and origami duck transformed from flat sheets.^[^ ^45^ ^]^ Copyright 2021, American Association for the Advancement of Science. g) The configuration of a cell and the layer‐by‐layer collapse of the metamaterials under uniaxial compression.^[^ ^172^ ^]^ Copyright 2019, Elsevier.

The SMPs themselves are not conductive, but they would become conductive after compounding with conductive fillers and then form continuous internal conductive networks. The characteristic of these composite materials is the obvious deformation stimulated by Joule heat when electrified.^[^ ^25^, ^166^ ^]^ Zhu et al.^[^ ^167^ ^]^ noticed that existing micro‐origami metamaterials did not respond in time and not programmable in shape. They designed electro‐thermal micro‐origami systems. The structures would achieve rapid, reversible elastic folding with large angles by controlling the input voltage. When overheated, there would be plastic folding and bringing reprogramming for the shape of the origami (**Figure** **8**a). Buckner et al.^[^ ^168^ ^]^ integrated functional fibers into the fabric to create a metamaterial robot, which was able to freely change its shape like a transformer (Figure 8b). Nitinol SMA wires were used as the bending actuators, and their flat ribbon section configuration avoided the uncontrollable and chaotic actions.

The electro‐responsive AMMs mentioned above conduct heat and deform by themselves. Some researchers combined the functions of “electro‐thermal effect” and “thermal deformation effect” via assigning functions to different materials in the laminates structure. He et al.^[^ ^61^ ^]^ made a sandwiched structure by compressing two layers of loosely cross‐linked LCEs and one layer of heating wires. The deformation could be accurately controlled by the applied electrical potential. And after curled, the metamaterials could be used as soft tubular actuators. Wang et al.^[^ ^169^ ^]^ designed biomimetic metamaterials by laminating three layers of materials: ultra‐thin deformation heater, Kapton film sensors with low thermal expansion coefficients, and carbon‐black‐doped liquid‐crystal elastomer (LCE‐CB) which would rapidly deform when heated, as shown in the Figure 8c. Since the thermal expansion coefficients of Kapton film and LCE‐CB were quite different, the metamaterials would deform due to mechanical mismatch under thermal effects. Moreover, by programming the internal micro‐architectures, the metamaterials could realize inchworm‐like movements under electrical stimuli. Xiao et al.^[^ ^170^ ^]^ reported another laminated metamaterial. Different from Wang et al., Xiao and coworkers replaced LCE‐CB with LCNs (liquid crystal networks) and expanded the shape morphing, mode of movements, and other functions of metamaterials (Figure 8d).

In addition, other kinds of electro‐responsive active metamaterials utilized the electrochemical effects.^[^ ^45^, ^165^, ^171^, ^172^, ^173^, ^174^ ^]^ Xia et al.^[^ ^171^ ^]^ employed alloying/dealloying reactions to electrochemically reconfigure the microstructures of the structures. Figure 8e shows the auxetic lattices during the electrochemically driven silicon‐lithium alloying reaction. The electrochemical oxidation reaction on the surface of the thin platinum metal film would produce strains leading to bend on the surface. Based on the similar principle, a nanoscale surface electrochemical actuator was prepared by Liu et al.^[^ ^45^ ^]^ And the actuators were assembled into micrometer‐scale origami quadruped robots and origami ducks (Figure 8f). Nick et al.^[^ ^172^ ^]^ set up micro‐fluidic channels filled with metal liquid inside the structural units of the metamaterials. When the structure was compressed or applied by forces, the microfluidic channel would deform and result in the change of the resistance of the metal liquid. Thus the smooth and continuous compression deformation of the buckling components of the metamaterials was converted into digital electrical signals. By assembling microstructure units of different configurations, the electrical conductivity and macro‐mechanical properties during loading and unloading cycles were programmable (Figure 8g). Li et al.^[^ ^35^ ^]^ applied electro‐responsive metamaterials to botany research, and they made conductive hydrogel metamaterials with ultra‐high adhesion. These metamaterials would attach to the uneven surfaces of hairy plants harmlessly, and effectively converted the mechanical signals into electrical signals.

### 3.5 Magneto‐Responsive Active Mechanical Metamaterials

Magnetorheological materials (MRMs) are mainstream magneto‐responsive materials due to their fast, continuous response and reversible morphological transformation. According to the type of matrix and the physical state of the materials, MRMs can be roughly divided into magnetorheological fluids (MRFs), magnetorheological elastomers (MREs), and magnetorheological gels, etc. Each type of MRMs has very different material behaviors, so they are suitable for different applications.

**Figure 9:**
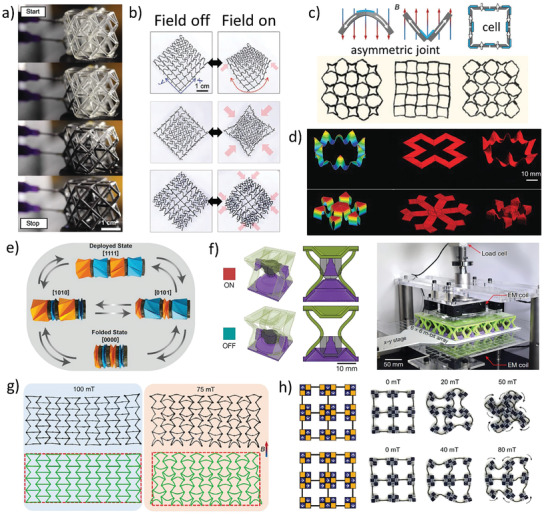
Magneto‐responsive active metamaterials. a) The infilling process of MRFs into cuboctahedron lattices.^[^ ^27^ ^]^ Copyright 2018, American Association for the Advancement of Science. b) Three typical deformation patterns of the magnetic meshes.^[^ ^178^ ^]^ Copyright 2019, Wiley‐VCH. c) Deformation under the magnetic field of asymmetric joints and the metamaterials array composed of cells with asymmetric joints.^[^ ^29^ ^]^ Copyright 2020, Wiley‐VCH. d) The deformations of the samples fabricated by DIW.^[^ ^179^ ^]^ Copyright 2018, Nature Publishing Group. e) Several programmable folding modes of Kresling origami assemblies.^[^ ^180^ ^]^ Copyright 2020, National Academy of Sciences. f) The ON and OFF state of the m‐bit bi‐stable cell and the tiled array with programmable mechanical properties.^[^ ^181^ ^]^ Copyright 2021, Nature Publishing Group. g) From left to right: the deformation of the M‐SMPs structures at 22 and 90 °C, with an upward magnetic field.^[^ ^182^ ^]^ Copyright 2020, American Physical Society. h) Metamaterials assembled by micro‐magnetic quadrupole modules.^[^ ^183^ ^]^ Copyright 2019, American Association for the Advancement of Science.

In 1948, an American engineer Jacob Rabinow^[^ ^175^ ^]^ found that the mixture of magnetic particles and water or oil tended to solidify when approaching the magnetic field. This is the first time that human discovered the magnetorheological effect. But in the early stage, the sedimentation of high‐density magnetic particles in MRFs hindered the development of magnetorheology. Afterward in 1990s, the introduction of some additives basically solved this problem, which led to the revival of MRFs. Currently, MRFs generally contain three components: Micron or nanoscale ferromagnetic particles, non‐magnetic liquid matrix, and some stabilizing additives. Under an external magnetic field, MRFs will rapidly change from the liquid state (which is similar to the Newtonian fluid) to the solid state. And the iconic spikes will form on the free surface of MRFs.^[^ ^176^ ^]^ During the process, the MRFs’ shear yield strength, apparent viscosity and storage modulus will rapidly increase within a few microseconds. After removing the magnetic field, MRFs can quickly return to their original liquid state. The generally accepted principle is that the magnetic particles in MRFs are magnetized into dipoles under the action of the external field, and the dipoles attract each other to arrange like a chain along the flux lines of the magnetic field.^[^ ^177^ ^]^ This accounts for the “solidification” phenomena and the increase in shear yield strength of MRFs. Utilizing the magnetorheological effect, Jackson et al.^[^ ^27^ ^]^ designed hollow cuboctahedron lattices infilled with MRFs (**Figure** **9**a). Then the effective stiffness could be efficiently modulated by tuning the applied magnetic field, and the stiffness could increase 35% when exposed to the magnetic field of 0.11 T. In general, the AMMs based on MRFs are not very in common. In the author's opinion, the main problem is that liquid MRFs cannot form a fixed shape without solid frameworks. Thus they can hardly be combined with the mechanical metamaterials configurations.

Different from MRFs, MREs are prepared by curing the mixture of magnetic particles and matrix materials, and hard to achieve the state transition and tremendous changes of mechanical properties like MRFs. Wu et al.^[^ ^184^ ^]^ have done a comprehensive review of the progress in the multifunctional MREs. But they did not particularly concern about the works of metamaterials in the article. The MREs are composed of soft elastomer matrix and magnetic particles. When the actuation magnetic forces are sufficient to overcome the restoring torque of the elastomer itself, the material can be actuated.^[^ ^185^ ^]^ Therefore, some relatively soft and compliant elastomers such as polydimethylsiloxane (PDMS) or Eco‐Flex are often selected as the matrix. The magnetic particles mainly include soft magnetic particles and hard magnetic particles. Normally, the hard magnetic particles can retain some magnetization (related to the specific type of magnetic particles and magnetization approach) when the external magnetic field is removed, while the soft magnetic particles cannot. Due to their different magnetic properties, the actuation principle of the MREs composed of soft magnetic particles are quite different from those composed of hard magnetic particles. And correspondingly, the two types of MREs have their own set of fabrication methods and scopes of application.

Soft magnetic particles that commonly used in magneto‐responsive metamaterials include Fe, Fe_3_O_4_, and carbonyl iron powder. They have relatively high magnetic permeability and low coercivity compared to the hard magnetic particles. The MREs composed of soft particles are essentially actuated by magnetostriction. Under the magnetic field, the soft magnetic particles in MREs are easily magnetized and tend to change from the original random rotation to be along the magnetic field. These interactions between the magnetic particles elongate the material, which is called magnetostriction.^[^ ^186^ ^]^ The resulting magnetic force per volume could be expressed as^[^ ^184^ ^]^

(1) F=∇B·M

where ∇*B* is the gradient of the magnetic flux density, and *M* is the magnetization of the materials. Roh et al.^[^ ^178^ ^]^ employed DIW process to print magneto‐responsive active metamaterials with the shape of “elastin‐like” meshes. The matrix of the AMMs was PDMS, and the magnetic parts were 4 µm carbonyl iron particles. These soft magneto‐responsive meshes would float on the water and reversibly shrink and expand as programmed under the magnetic field (Figure 9b). In order to enhance the magneto‐responsive effect, an external magnetic field is usually applied during the crosslinking process, so that the soft magnetic particles can be arranged in a chain‐like or pillar‐like form along the direction of the magnetic field until cured. Kim et al.^[^ ^187^ ^]^ proposed that the easy axes of a magnetically anisotropic material are the energetically favorable directions of the magnetization. They fabricated the micro‐actuators, which were assembled from multiple components with different easy axes. The products were able to bend or crawl in the preset way controlled by the magnetic field. Mishra et al.^[^ ^188^ ^]^ prepared the cross shaped MRE films from Fe_3_O_4_ particles and thermoplastic polyurethane (TPU). They found that whether the internal magnetic particles were uniformly dispersed or arranged in chains, had an obvious impact on the MREs’ selective response of the applied magnetic field. Although the anisotropy could be achieved by some treatments, MREs made from soft magnetic particles are generally are not as efficient as those prepared with hard magnetic particles.

MREs composed of hard magnetic particles are new magneto‐responsive materials which has attracted much attention recently.^[^ ^189^ ^]^ They could maintain magnetization when the applied magnetic field is removed due to the high remanence and large coercivity. MREs made of hard magnetic particles are manipulated by the magnetic torque, which is generated when the magnetization of the hard magnetic particles are not in line with the applied magnetic field.^[^ ^185^, ^190^ ^]^ The magnetic torque represents the tendency to align the MREs’ magnetization direction with the external magnetic field to lower the energy of the material systems. The torque vector ****τ**** per volume could be expressed as^[^ ^184^ ^]^

(2) τ=M×B

where *M* is the magnetization of the materials, and *B* the magnetic flux density. During the preparation process, some magnetization distributions within the MREs are usually pre‐programmed by permanent magnet or pulse magnetizers. Therefore, a series of complicated and reversible shape transformations of the productions could be controlled by the external magnetic field. The most frequently used hard‐magnetic particles are NdFeB particles. Gu et al.^[^ ^28^ ^]^ developed magnetic soft robots with programmable metachronal waves. They programmed the magnetization direction of the magnetic cilia carpets through rolling them up and exposing them to the impulse magnetizer. With the actuation of an external dynamic magnetic field, the carpets had capabilities to achieve motion modes of flow transportation, crawling, and rolling like a millipede robot. Cui et al.^[^ ^190^ ^]^ developed four types of nanomagnet cells with different hysteresis loops, and connected the some of these cells together with soft hinges to fabricate various configurations. Under the non‐uniform magnetic field, the response of each step of each part would be encoded, and the original 2D structures would show complex 3D actions. The highlight of this work is that they could realize the manipulation on nanomagnets to a nanometer scale. Wu et al.^[^ ^191^ ^]^ and Montgomery et al.^[^ ^29^ ^]^ designed a novel asymmetric joint, which made the network structures deform with multiple deformation modes under the opposite direction of magnetic field (Figure 9c). Kim et al.^[^ ^179^ ^]^ reported a DIW concept, which meant designing the magnetic density and direction distribution through magnetization while printing, and directly producing programmed structures. This fabrication expanded the design freedom, and they had made AMMs that can perform complex actions driven by magnetic fields (Figure 9d). Inspired by kresling origami, Novelino et al.^[^ ^180^ ^]^ designed functional microrobots, which had magneto‐responsive plates on the free end and copper tapes attached inside. The magneto‐responsive plates were composed of NdFeB. As the torque theory mentioned before, there would be a magnetic torque to align the magnetization direction of the plate with the direction of the external magnetic field, and thereby driving the folding and deploying of each origami cell independently. As a result, the copper tapes circuit would be on and off, and then allowing for on‐the‐fly programmability (Figure 9e). Chen et al.^[^ ^181^ ^]^ utilized MREs to design the reprogrammable multi‐stable metamaterials. They developed a bi‐stable elastic structure cell, which consists of a magnetic cap made from the NdFeB particles and a resin framework, as shown in Figure 9f. Controlled by the external magnetic field, the cell would be adjusted to ON (high stiffness modulus state) and OFF (low stiffness modulus state) states, which corresponded to a binary element (m‐bit) of 1and 0. Then the mechanical properties of the tiled array would be programmed on demand. At present, AMMs based on the hard magnetic particles emerge incessantly. Combing with the SMPs, Ma et al.^[^ ^182^ ^]^ prepared the magnetic shape memory polymers (M‐SMPs). The deformations of the M‐SMPs could be controlled by both magnetic field and the thermal field. They designed a variety of hourglass configurations with tunable Poisson's ratio and bending deformation (Figure 9g), which could realize controllable, reprogrammable shape transformation and shape locking. The MREs made from hard magnetic particles show broad prospects in biomedicine, microfluidics, and microrobots, which makes it one of the most promising development fields in the future.

When the size of the ferromagnetic particles is as small as nanoscale, it would be heated by the high frequency alternating magnetic field. And this effect is widely used for hyperthermia therapy and tissue engineering. Zhang et al.^[^ ^192^ ^]^ proposed a simple 4D printing strategy: mixing the magnetic composite filament (Fe_3_O_4_+ PLA (polylactic acid)) and the pure PLA filament to printing magneto‐responsive structures. Under an alternating magnetic field of frequencies from 27.5 to 47.5 kHz, the magnetic nanoparticles in composite structures would be triggered to vibrate and generate heat, resulting in the deformation of the SMP matrix. After this, Zhao et al.^[^ ^193^, ^194^ ^]^ utilized the fabrication technology again to design porous scaffold structures similar to lotus root, which were hopefully to be used for the repair and regeneration of human tissues. The above three works essentially belong to the thermal‐response category. Ze et al.^[^ ^195^ ^]^ proposed the concept of another M‐SMPs metamaterials, which contained the soft magnetic particles Fe_3_O_4_ and the hard magnetic particles NdFeB. Different from the M‐SMPs of Ma et al.,^[^ ^182^ ^]^ the heat in this work was generated by the inductive heating of the Fe_3_O_4_ particles by applying a high frequency alternating current (AC) magnetic field. Their design saved the trouble of providing an extra thermal field.

Utilizing the natural magnetic forces of the small permanent magnets, researchers also developed simple but novel AMMs. Gu et al.^[^ ^183^ ^]^ introduced external magnetic field to excite the magnetic forces and the actuation process was continuous. They proposed a concept of magnetic quadrupole module that can be assembled to form arbitrary 2D shapes with arbitrary magnetizations. To demonstrate this design strategy, they developed the auxetic structures with soft ligaments. As shown in Figure 9h, the dynamic deformation of the soft structures could be programmed by the applied magnetic field.

### 3.6 Pressure‐Responsive Mechanical Metamaterials

Due to the advantages of convenience and reusability, the pressure actuation method has been widely used for AMMs. Setting up channels or cavities inside the soft matrix and changing the pressure through inflation or deflation (filling or draining) would change the shapes or locomotions of the metamaterials. Pneumatic actuation is one of the most popular pressure‐driven approaches.

#### 3.6.1 Pneumatic Actuation Active Mechanical Metamaterials

**Figure 10:**
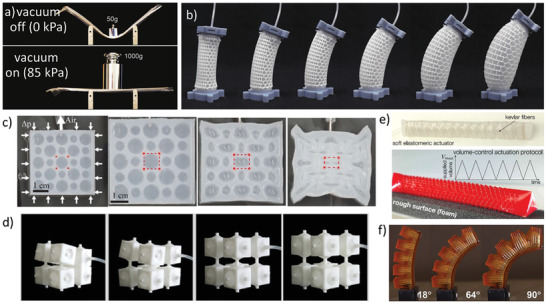
Pressure‐responsive metamaterials. a) The bearing capacity of the laminated structures with the change of internal pressure.^[^ ^196^ ^]^ Copyright 2018, Wiley‐VCH. b) Deformed states of the bending metamaterial under pressures from 0 to 0.025MP.^[^ ^197^ ^]^ Copyright 2020, Springer Nature. c) The deformation of the square lattices with alternatively arranged holes of different sizes.^[^ ^30^ ^]^ Copyright 2020, Elsevier. d) Specimen and the deformation process of the pneumatic negative stiffness metamaterials.^[^ ^198^ ^]^ Copyright 2020, Elsevier. e) The construction details of the inner pneumatic actuator and kirigami skin.^[^ ^97^ ^]^ Copyright 2018, American Association for the Advancement of Science. f) Schematics of the hydraulic actuated autonomic perspiration robot fingers.^[^ ^204^ ^]^ Copyright 2020, American Association for the Advancement of Science.

Narang et al.^[^ ^196^ ^]^ proposed a soft metamaterial (**Figure** **10**a) inspired by the laminar jamming phenomenon, which implied that a laminate of compliant strips would become strongly coupled through friction when a pressure gradient was applied and resulted in the changes of its mechanical properties. In this study, an acrylic frame enclosing copy paper sheets and a vacuum tube were fabricated, and its mechanical properties could be easily controlled by regulating its inner pressure by inflation or deflation. Combining the pneumatic actuation principle with the common metamaterials design schemes would realize real‐time adjustment effects. Pan et al.^[^ ^197^ ^]^ utilized a pneumatic rubber tube and metamaterials networks to make a new soft bending metamaterial. As Figure 10b shows, auxetic and non‐auxetic structures, corresponding to positive Poisson's ratio and negative Poisson's ratio, were deployed on both sides of the pneumatic tube and enveloped. The mechanical properties of the coupled structure were pre‐designed by arranging the distribution of the two kinds of materials. Chen et al.^[^ ^30^ ^]^ designed a kind of pneumatically actuated metamaterials with periodically arranged and interconnected holes. The stiffness, Poisson's ratio, and critical condition for the pattern transformation could be tuned by pneumatically changing the shape of the holes (Figure 10c). Similarly, Tan et al.^[^ ^198^, ^199^ ^]^ developed a novel real‐time tunable negative stiffness mechanical metamaterials consisted of urethane elastomer cavities that were able to inflate like balloons (Figure 10d). Uniaxial compression and vibration tests were performed to reveal the influence of the inner pressure on its mechanical properties. In addition, origami and kirigami structures are particularly suitable for combining with pneumatic actuation methods due to their good deployable and foldable properties. Li et al.^[^ ^200^ ^]^ reported a soft gripper made of a vacuum‐driven origami. The gripper could easily open and contract by pneumatical control. Owing to its soft skin and skeleton, the gripper could safely grasp targets with various shapes. Also based on origami, Kim et al.^[^ ^201^ ^]^ were inspired by the pelican eel's predation action and they designed a “dual‐mode” stretchable origami. In this study, two elastomers with different stiffness were patterned into each side of the flexible origami. As inner pressure rising, the robot's morphing mode transformed from a geometric‐dominant mode to a strain‐dominant mode, which resulted in a secondary morphing. Besides, Rafsanjani et al.^[^ ^31^, ^97^ ^]^ proposed a novel bionic snake robot, whose body was made of a single pneumatic actuator and skin made of stretchable kirigami (Figure 10e). When inflated, the body stretched; when deflated, the robot's kirigami skin gripped the substrate on some anchoring points to pull itself forward meanwhile prevent backward sliding. By alternately inflating and deflating to control the expansion and contraction of the robot, it would crawl as the locomotion of the snakes. Different from Rafsanjani, Shepherd et al.^[^ ^202^ ^]^ utilized five independent pneumatic actuators to make a crawling soft robot. They employed pneu‐nets (PNs) metamaterials, which consisted of series of chambers fixed on an elastomer substrate attached to an inextensible layer, to realize low‐pressure pneumatic actuation and rapid response. Four PNs were used as limbs to allow crawling, and one PN as the main body to achieve lifting and curling movements, so the robot could adjust its gaits to easily cross obstacles. Based on kirigami, Babaee et al.^[^ ^203^ ^]^ designed a stent based on the pneumatic actuation method. Although their work had been applied for targeted drug delivery and some other functions, it still had the problems of connecting the catheter and continuous pressure input, and which restricted the structure's flexibility. Therefore, applying pneumatic actuation approaches to millimeter‐scale or in complex environments still seems to be challenging tasks.

#### 3.6.2 Hydraulic Actuation Active Mechanical Metamaterials

Hydraulic actuated active metamaterials and pneumatic actuated active metamaterials are similar in principle, the main difference between them is that the fluid is more viscous and less compressible than air.^[^ ^205^ ^]^ In practical, most pneumatic‐driven cases can be substitute with hydraulic‐driven methods.^[^ ^206^ ^]^

Li et al.^[^ ^207^ ^]^ reported a 3D printed fluidic origami consisted of rigid facets and rubber creases. In this study, the stiffness of the origami could be tuned by the volume of fluid filled in it. Li et al.^[^ ^208^ ^]^ proposed a kind of artificial muscle made of fluid‐driven origami, in which shells were TPU‐coated nylon fabrics and the zigzag skeletons nylon plates. When removing the fluid in the artificial muscle, it would contract and be able to make movements like lifting heavy objects. Considering the coupling with other physical fields when designing pressure‐driven metamaterials may obtain unexpected excellent performance. Mishra et al.^[^ ^204^ ^]^ reported a kind of novel hydraulic actuated autonomic perspiration robot fingers (Figure 10f). The localized pores within the hydrogel fingers resulted in a smart deformation mode: when below 40 °C, the pores were closed and the robot fingers’ motions would be manipulated by hydraulic actuation; when it was 40 °C or more, the pores would dilate to perspiration, thereby adjusting the grip strength and rapidly cool down.

### 3.7 The Pros and Cons of the Active Mechanical Metamaterials with Different Stimuli Fields

From the works above, there are huge differences between the various stimuli‐responsive materials. 1) The thermal‐responsive materials are the most widely used for AMMs due to their convenient operation and tunable temperature. Large amounts of applications based on thermal‐responsive AMMs have been developed for deployable structures, flexible electronics, biomedicine, and many other fields. Nevertheless, the thermal stimuli were mainly obtained from the external environment, making the response relatively slow to several seconds or even a few minutes. Moreover, at present, providing an accurate gradient temperature field is not easy. Thus the precise controlling of the specific components of the structures could be hard to realize. 2) For the chemical‐responsive materials, the precise control and sensitive response make them suitable for miniaturized systems. It should be noted that diverse stimuli‐responsive material selections would lead to great differences. Both based on the swelling principle, the response speed of Wei et al.’ work^[^ ^145^ ^]^ is obviously slower than Li et al.’s work.^[^ ^209^ ^]^ Besides, some of the chemical‐responsive materials are expensive or quickly consumed, and they can only be performed under laboratory conditions. 3) The light‐responsive AMMs generally have advantages of timeliness, remote and precise control. And light sources such as near‐infrared are able to penetrate the skin but without damage to human tissues. Therefore, some kinds of light‐responsive AMMs can be used in biomedicine. 4) Electro‐responsive materials are safe and easy to achieve. However, quite part of the metamaterials based on electro‐thermal effect need to be connected by wires. Thus they are inconvenient when compared with the metamaterials based on thermal‐responsive or photo‐thermal effect. This major defect may become the direction for researchers to improve in the future. Among the works of AMMs in recent years, electro‐responsive AMMs are relatively less to be used. 5) Magneto‐responsive AMMs are emerging metamaterials receiving much attention in recent decades. They have superior characteristics like quick response, contactless and continuous control, low energy consumption, and are harmless to the human tissues. Besides, the magnetic field travels through the non‐magnetic medium easily and undisturbed, so it would be suitable for implement the tasks that need to be operated in an enclosed space such as the human body. Due to the advantages mentioned above, Magneto‐responsive AMMs will be the best choice in the field of biomedicine. The disadvantages are the complex facilities and stringent experimental conditions. And if applied to large‐scale equipment, it often requires a much higher cost than other actuation methods. 6) Pressure‐responsive AMMs are easy to implement and do not require complicated experimental environment. Just an air pump with stable pressure output would meet the conditions of most pressure‐responsive AMMs. The main shortcoming is the poor accuracy of control, and hardly to be applied to the micro‐structure systems.

For the convenience of follow‐up researches to select the corresponding stimulus response materials and design strategies according to the needs, we compare some representative AMMs of different stimuli fields, as is shown in **Tables** **1** and **2**. (“‐” means the properties are not mentioned or could not be calculated from the contents.)

**Table 1:** Comparison of the various stimuli‐responsive AMMs (Part I)

Material system	Actuation method	Activation time	Required energy	Maximum deformation/strain	Characteristic size	Structural configuration	Refs
Monomers/crosslinker	Heat	–	≈122 J	50%	30 mm	Kresling origami	^[^ ^37^ ^]^
Photopolymer (SMPs)	Heat	–	≈160 J kg^−1^ at 30 °C	≈70%	40 mm	Kresling origami	^[^ ^38^ ^]^
Polymer PLA	Heat	–	≈100 J	95%	120 mm	Network stent	^[^ ^119^ ^]^
Paper and SMAs	Heat	120 s	–	−40–10%	90 mm	Origami	^[^ ^128^ ^]^
Polymer	Heat	90 s	–	–	80 mm	Lattices	^[^ ^138^ ^]^
Hydrogels	Chemical	60 s	–	≈127%	10 mm	Squares	^[^ ^143^ ^]^
Hydrogels	Chemical	120 min	–	*x*: 18.8%, *y*: 21.6%	30 mm	Lattices	^[^ ^145^ ^]^
Polymer	Chemical	12 s	–	–	100 µm	Lattices	^[^ ^209^ ^]^
Rubber and light curing resin	Light	≈1.87 s	6 J	–	50 mm	Origami	^[^ ^24^ ^]^
Resin	Light	4–30 s	10–25 mW cm^−2^	–	≈20 mm	Origami	^[^ ^163^ ^]^
Polymer thin sheet	Light	8–14 s	–	–	≈70 mm	Origami	^[^ ^164^ ^]^
Graphene nanoplatelets (GNPs) and polycaprolactone (PCL)/polyurethane (PU)	Electricity	≈40 s	20 mW cm^−2^	≈100%	20 mm	Curved shape	^[^ ^25^ ^]^
Hydrogel and PDMS	Electricity	1.3 s	1e‐5 W	135%	3 mm	Biohybrid patch	^[^ ^35^ ^]^
SU‐8	Electricity	–	3.0 V of voltage and 20 mW of power	≈90°	1 mm	Origami	^[^ ^167^ ^]^
Hydrogels and SMPs	Electricity	90 s	–	≈50%	35 mm	Ripple shape	^[^ ^170^ ^]^

**Table 2:** Comparison of the various stimuli‐responsive AMMs (Part II)

Material system	Actuation method	Activation time	Required energy	Maximum deformation/strain	Characteristic size	Structural configuration	Refs
MRFs	Magnetic field	0.83 ± 0.05 s (field on), 0.23 ± 0.06 s (field off);	–	10% (0.11 T)	≈10 mm	Cuboctahedron lattices	^[^ ^27^ ^]^
4.2 µm carbonyl particles, PDMS	Magnetic field	<0.1 s	–	24%	36 mm	Networks	^[^ ^178^ ^]^
Soft polycrystalline ferromagnetic material, silicon nitride membrane	Magnetic field	Nanoseconds level	≈2.6e‐15J	–	20 µm	Four‐panel origami	^[^ ^190^ ^]^
25 µm NdFeB particles, SMPs	Magnetic field	–	–	≈110%	55 mm	Chiral networks	^[^ ^182^ ^]^
5 µm NdFeB particles, UV resin	Magnetic field	<0.1 s (7 mT)	–	–	4 mm	Multi‐armed microgripper	^[^ ^210^ ^]^
Polyester plastic sheets	Pneumatic actuation	–	16 KJ m^−3^	20%	40 mm	Kirigami	^[^ ^31^ ^]^
Plastic sheet	Pneumatic actuation	10 s	–	25%	32 cm	Kirigami	^[^ ^97^ ^]^
Silicone rubbers	Pneumatic actuation	–	50 KJ m^−3^	5%	80 mm	PPR and NPR	^[^ ^197^ ^]^
Urethane elastomer and gelatum	Pneumatic actuation	–	25 mJ	90%	37 mm	Disk structure	^[^ ^199^ ^]^
Polyether ether ketone and PVC film	Hydraulic actuation	0.2 s	2000 W kg^−1^	50%	19 cm	Origami	^[^ ^208^ ^]^

## 4 Functions and Practical Applications of Active Mechanical Metamaterials

### 4.1 Functions of Active Mechanical Metamaterials

The extraordinary mechanical properties of mechanical metamaterials (or structural metamaterials) derive from artificial microstructures in 3D space. Most of mechanical metamaterials are related to the basic mechanical parameters of Young's modulus *E*, shear modulus *G*, bulk modulus *K*, and Poisson's ratio *ν*, and those parameters are used to measure the stiffness, rigidity and compressibility of the productions. When combined with stimuli‐responsive materials, their functions will be further expanded. A series of new functions represented by active shape‐shifting have been developed.

#### 4.1.1 Active Shape‐Shifting

Active shape‐shifting is the most widely used function of AMMs and has been involved in almost every section of this review. Based on this, the features such as multistability, mode conversion, reconfigurability, and structural deployablility could be achieved. In essence, active shape‐shifting metamaterials are similar to the critical state of the buckling of the column under axial loading. Where reversible elastic instability state causes the positive or negative changes of the equivalent elastic modulus of the whole structure. Active shape‐shifting metamaterials could be roughly classified as two levels. The first is that the components are activated by the stimuli fields and directly deform or make action responses.^[^ ^35^, ^45^, ^163^ ^]^ This helps to achieve untethered control. The second is to change the mechanical properties like Young's modulus through external stimuli fields,^[^ ^27^, ^37^, ^38^, ^39^, ^138^, ^196^ ^]^ and the process could be reversible within certain ranges.

**Figure 11:**
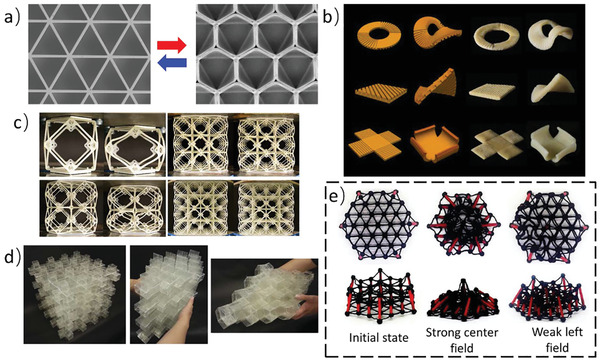
Functions of active mechanical metamaterials. a) Top view of the initial triangular micro‐cellular structures and assembled micro‐cellular structures.^[^ ^209^ ^]^ Copyright 2021, Nature Publishing Group. b) Three morphing pasta shapes of initial and cooked state. From top to bottom are ring, saddle, box.^[^ ^211^ ^]^ Copyright 2021, American Association for the Advancement of Science. c) Metamaterial voxels and the multi‐unit modules.^[^ ^46^ ^]^ Copyright 2020, American Association for the Advancement of Science. d) Origami metamaterials with functions of shapes reconfigurable and acoustic waveguides.^[^ ^213^ ^]^ Copyright 2016, American Association for the Advancement of Science. e) Contraction and deployment of the auxetic tensegrity actuator under strong center magnetic field and weak left field.^[^ ^44^ ^]^ Copyright 2020, American Association for the Advancement of Science.

New achievements still emerge incessantly. Based on the swelling mechanism, Li et al.^[^ ^209^ ^]^ designed an innovated chiral honeycomb micro‐scale metamaterial. When a drop of liquid was added, the capillary forces generated from the evaporation of liquid would change the triangular lattices into hexagonal lattices (**Figure** **11**a), and the process was stably reversible. Inspired by the various shapes of spaghetti, Tao et al.^[^ ^211^ ^]^ designed series of swelling metamaterial with surface grooves. The uneven diffusion in the center and surrounding areas of the structures would induce rapid shape‐shifting. Only tuning the shapes of the grooves could make them change into ring, saddle, or box after swelling (Figure 11b). Yang et al.^[^ ^212^ ^]^ developed kirigami grippers, which buckled under external tension. But the process could only be operated by manual operations or complex manipulators. Yang and coworkers induced magneto‐responsive materials, which enabled precise control for the motions of kirigami grippers.

#### 4.1.2 Load Bearing and Impact Protection

Load bearing and impact protection are other important functions of AMMs. These kinds of metamaterials are always with lightweight, high stiffness/strength, and have been applied to fields ranging from precision parts manufacturing to giant aircraft airfoils. Qin et al.^[^ ^214^ ^]^ utilized minimal surfaces to fabricate a macroscopic lightweight 3D graphene assembly. This kind of metamaterial could retain its strength even in extreme conditions. Bauer et al.^[^ ^215^ ^]^ proposed mechanical metamaterials with integral self‐tensioning truss architectures, which were both ultralight and failure‐resistance. Jenett et al.^[^ ^46^ ^]^ put forward the concept of discretely assembled mechanical metamaterials, which was like Lego building blocks (Figure 11c). Single metamaterial voxels with different mechanical properties (such as rigidity, compliance, auxeticity, and chirality) were assembled. The assembled metamaterials were impact resistant and distorting when compressed to reduce the damage. And they finally put this idea into a voxel racing car. In recent decades, braided metamaterials with lightweight features, high out‐of‐plane stiffness, impact protection function, and damage tolerance are popular for aerospace and transportation. Moestopo et al.^[^ ^216^ ^]^ fabricated braided metamaterials with the capability for multiple tension and compression cycles. Utilizing finite element simulation, some researchers demonstrated the mechanical behavior of 3D braided composites under tensile load^[^ ^217^ ^]^ and the conditions with defects.^[^ ^218^, ^219^, ^220^ ^]^

#### 4.1.3 Elastic Waves Propagation Adjustment

Mechanical metamaterials also have the function of adjusting the propagation of elastic waves and controlling the propagation of acoustic waves. An et al.^[^ ^221^ ^]^ proposed 3D truss‐lattice metamaterials, which had a good vibration suppression effect for low‐frequency and broad‐band elastic waves. The main principle was that the local resonance mechanism produced due to the body centered cubic lattices prevented the elastic waves to pass through. Zhang et al.^[^ ^40^ ^]^ utilized the swelling effect of hydrogel to fabricate soft network metamaterials with tunable configuration. Compared with the initial state, the fully hydrated configuration exhibited obvious phononic wave band gaps. Ren et al.^[^ ^41^ ^]^ designed multi‐stable metamaterials to achieve elastic wave bandgaps tuning. The tuning process was temperature‐induced due to the use of thermal responsive components. Subsequently, Wei et al.^[^ ^222^ ^]^ used chiral auxetic configurations to realize similar functions, and the advantage was that the specific elastic waves propagation mode could be fixed without continuous energy consumption. The AMMs above‐mentioned utilized the shape transformations of the structures to control the frequency of sound waves or elastic waves. But the real‐time control is hard for them. Combing magneto‐responsive materials with the lattices configuration, Yu et al.^[^ ^223^ ^]^ prepared novel magneto‐responsive acoustic metamaterials. The shape of the AMMs could be tuned by the remote magnetic field, so that they would be able to switch their effective constitutive parameter pairs among double‐positive, single‐negative, and double‐negative modes. The highly tunable constitutive parameters of these magneto‐responsive AMMs bring a new concept for acoustic devices.

#### 4.1.4 Acoustic Stealth

Pentamode metamaterials have five zero eigenvalues of modulus matrices,^[^ ^224^ ^]^ and the shear modulus *G* is much smaller than the bulk modulus *K*, which brings unique elasticity. Pentamode metamaterials are also called shear modulus weakened metamaterials, the mechanical properties are similar to the 2D ideal fluid. The function in acoustic control of pentamode metamaterials was first raised by Norris et al.,^[^ ^225^ ^]^ and this kind of control would be evolved into applications of acoustic stealth cloaks.^[^ ^4^, ^226^ ^]^ The typical cell of pentamode metamaterials^[^ ^3^ ^]^ consisted of four biconical elements that connected at one point. Huang et al.^[^ ^5^ ^]^ experimented with a variety of section shapes for regular triangle, square, pentagon, hexagon, and circle. The regular triangle section turned out to be with the best acoustic stealth performance through simulation analysis. Pentamode metamaterials achieve the function of not only acoustic stealth. Xue et al.^[^ ^227^ ^]^ designed an underwater acoustic cloak using soft metamaterials with Poisson's ratio of −1. It was self‐adaptive because the quasi‐conformal mapping requirement could be automatically satisfied. Both Chen et al.^[^ ^228^ ^]^ and Ning et al.^[^ ^229^ ^]^ made acoustic bandgap adjustment designs based on chiral configurations. Besides, Babaee et al.^[^ ^213^ ^]^ innovatively used the 3D origami configuration to guide the acoustic wave propagation. The networks of tubes of the origami metamaterials could be flexibly adjusted by folding and unfolding the origami (Figure 11d).

#### 4.1.5 Tunable Expansions

The typical NTE behavior is that the whole metamaterial shrinks in one or more directions when it is heated, that is, the coefficient of thermal expansion is negative. In AMMs, NTE are usually obtained by combining two or more materials with different thermal expansion coefficients. By adjusting the combination of materials or the curvature of the structures, various metamaterials with adjustable thermal expansion coefficients can be obtained. It has been discussed in Sections 2.1.2^[^ ^11^, ^12^, ^13^, ^14^, ^15^, ^16^, ^17^, ^18^, ^19^, ^20^, ^21^, ^22^, ^23^, ^24^, ^25^, ^26^, ^27^, ^28^, ^29^, ^30^, ^31^, ^32^, ^33^, ^34^, ^35^, ^36^, ^37^, ^38^, ^39^, ^40^, ^41^, ^42^, ^43^, ^44^, ^45^, ^46^, ^47^, ^48^, ^49^, ^50^, ^51^, ^52^, ^53^, ^54^, ^55^, ^56^, ^57^, ^58^, ^59^, ^60^, ^61^, ^62^, ^63^, ^64^, ^65^, ^66^, ^67^, ^68^, ^69^, ^70^, ^71^, ^72^, ^73^, ^74^, ^75^ ^]^ (the refs here should be [11,73‐74]) and 3.1.^[^ ^20^, ^128^, ^139^ ^]^ It should be noted that here we regard the NTE metamaterials as mechanical metamaterials rather than thermal metamaterials. Because they are classified according to the mechanism of structural design. The heat source is only a condition to drive the deformation but not the parameter we control.

#### 4.1.6 Mobility

Recently, tensegrity metamaterials have attracted extensive attention due to their multiple functions and excellent mechanical properties. Typical tensegrity metamaterials are composed of rigid struts and flexible cables,^[^ ^43^ ^]^ where rigid struts only bearing compression and the flexible cables only bearing tension. Tensegrity metamaterials have unique high mobility, scalability,^[^ ^230^, ^231^ ^]^ which could be utilized to make mobile robots.

Researchers have developed many interesting smart robots by combining tensegrity configurations with thermal‐responsive, light‐responsive, and magneto‐responsive materials. Liu et al.^[^ ^42^ ^]^ utilized SMPs to design programmable tensegrity structures. Under the thermal excitation, the SMP struts would recover to straight shapes, and the whole structure deployed as a spherical tensegrity. Wang et al.^[^ ^43^ ^]^ used light‐responsive LCE‐CNT composite as cables and stiff plexiglass as struts, to make smart tensegrity robot. Through stimulating the cables by light selectively, the robot could easily roll, turn, carry objects and cross multiple kinds of terrain. Lee et al.^[^ ^44^ ^]^ mixed magnetic particles into the soft matrix to fabricate cables so that the movements of the tensegrity robot could be flexibly controlled through external magnetic fields (Figure 11e).

### 4.2 Practical Applications of Active Mechanical Metamaterials

The engineering applications of AMMs range from nano scaled micro‐machine to space shuttle. They are listed as follows. 1) The applications in smart robots include crawling robots,^[^ ^31^, ^97^, ^202^, ^210^, ^232^, ^233^, ^234^, ^235^, ^236^ ^]^ swimming robots,^[^ ^237^, ^238^, ^239^ ^]^ smart grippers,^[^ ^125^, ^201^, ^212^, ^240^, ^241^, ^242^ ^]^ smart wearable systems.^[^ ^27^, ^52^, ^168^, ^243^ ^]^ 2) The applications in miniaturized systems include micro actuators,^[^ ^166^, ^197^, ^244^, ^245^, ^246^, ^247^, ^248^, ^249^ ^]^ soft pumps,^[^ ^250^, ^251^, ^252^ ^]^ artificial muscles,^[^ ^204^, ^208^, ^253^, ^254^, ^255^ ^]^ microfluidics,^[^ ^28^, ^99^, ^256^ ^]^ super‐hydrophobic surfaces,^[^ ^257^ ^]^ smart sensors and electronic components,^[^ ^10^, ^53^, ^173^, ^258^, ^259^ ^]^ super‐capacitors,^[^ ^260^ ^]^ flexible and deformable batteries.^[^ ^51^, ^261^, ^262^ ^]^ 3) The applications in giant machinery, such as, aerospace, including smart load‐bearing and anti‐impact structures,^[^ ^1^, ^27^, ^46^, ^181^, ^263^ ^]^ morphing airfoils,^[^ ^264^ ^]^ acoustic stealth cloaks,^[^ ^48^, ^225^, ^226^, ^227^ ^]^ elastic mechanical cloaks,^[^ ^48^ ^]^ reconfigurable antenna,^[^ ^24^, ^180^, ^265^, ^266^ ^]^ terahertz metadevices,^[^ ^267^ ^]^ electromagnetic stealth system.^[^ ^268^, ^269^, ^270^ ^]^ 4) The applications in biomedicine include electronic skin,^[^ ^258^, ^271^, ^272^, ^273^, ^274^, ^275^ ^]^ tissue engineering,^[^ ^192^, ^194^, ^276^, ^277^, ^278^, ^279^, ^280^ ^]^ vascular stents,^[^ ^54^, ^136^, ^281^, ^282^, ^283^ ^]^ drug delivery carriers.^[^ ^136^, ^210^, ^284^, ^285^ ^]^ From the above discussion, the rich functions and application value of AMMs have brought great convenience to human production and life. And through the introduction of stimuli‐responsive materials, the applications of AMMs have been further expanded. It is envisioned that mechanical active metamaterials will play a more important role in the future.

## 5 Conclusion and Perspectives

The combination of the mechanical metamaterials and stimuli‐responsive materials produces AMMs, whose multiple controllable variables leading to an extensive range of applications. Based on current research, most AMMs exist in the form of multi‐material integration, in which elastic materials are the main support structure, and the stimulus materials are the sensing and driving components. Several substantive problems in the study of AMMs needed to be broken through urgently, including improving the properties of stimuli‐responsive materials and the collaborative design with elastic support materials. The metamaterial designs based on the characteristics of active materials also need new alternative materials and fabrication techniques. Moreover, in addition to superior mechanical properties and environmental sensing and driving functions, other special physical functions (such as, light, sound, electromagnetic, etc.) and biocompatibility should also be concerned to promote the continuous development and application of these new types of metamaterials. Advances in computer material science and additive manufacturing technology will significantly enhance the development of AMMs, and in the future, AMMs will play a more important role in all aspects of production and human life.

## Conflict of Interest

The authors declare no conflict of interest.
